# Evaluation of the safety profile of COVID-19 vaccines: a rapid review

**DOI:** 10.1186/s12916-021-02059-5

**Published:** 2021-07-28

**Authors:** Qianhui Wu, Matthew Z. Dudley, Xinghui Chen, Xufang Bai, Kaige Dong, Tingyu Zhuang, Daniel Salmon, Hongjie Yu

**Affiliations:** 1 School of Public Health, Key Laboratory of Public Health Safety, Fudan University, Ministry of Education, Shanghai, China; 2 Institute for Vaccine Safety, Johns Hopkins Bloomberg School of Public Health, Baltimore, USA; 3 Department of International Health, Johns Hopkins Bloomberg School of Public Health, Baltimore, USA; 4 Shanghai Institute of Infectious Disease and Biosecurity, Fudan University, Shanghai, China; 5 Department of Infectious Diseases, Huashan Hospital, Fudan University, Shanghai, China

**Keywords:** Novel coronavirus diseases 2019, Severe acute respiratory syndrome coronavirus 2, Vaccine, Safety profile, Review

## Abstract

**Background:**

The rapid process of research and development and lack of follow-up time post-vaccination aroused great public concern about the safety profile of COVID-19 vaccine candidates. To provide comprehensive overview of the safety profile of COVID-19 vaccines by using meta-analysis technique.

**Methods:**

English-language articles and results posted on PubMed, Embase, Web of Science, PMC, official regulatory websites, and post-authorization safety surveillance data were searched through June 12, 2021. Publications disclosing safety data of COVID-19 candidate vaccines in humans were included. A meta-analysis of proportions was performed to estimate the pooled incidence and the pooled rate ratio (RR) of safety outcomes of COVID-19 vaccines using different platforms.

**Results:**

A total of 87 publications with safety data from clinical trials and post-authorization studies of 19 COVID-19 vaccines on 6 different platforms were included. The pooled rates of local and systemic reactions were significantly lower among inactivated vaccines (23.7%, 21.0%), protein subunit vaccines (33.0%, 22.3%), and DNA vaccines (39.5%, 29.3%), compared to RNA vaccines (89.4%, 83.3%), non-replicating vector vaccines (55.9%, 66.3%), and virus-like particle vaccines (100.0%, 78.9%). Solicited injection-site pain was the most common local reactions, and fatigue and headache were the most common systemic reactions. The frequency of vaccine-related serious adverse events was low (< 0.1%) and balanced between treatment groups. Vaccine platforms and age groups of vaccine recipients accounted for much of the heterogeneity in safety profiles between COVID-19 vaccines. Reporting rates of adverse events from post-authorization observational studies were similar to results from clinical trials. Crude reporting rates of adverse events from post-authorization safety monitoring (passive surveillance) were lower than in clinical trials and varied between countries.

**Conclusions:**

Available evidence indicates that eligible COVID-19 vaccines have an acceptable short-term safety profile. Additional studies and long-term population-level surveillance are strongly encouraged to further define the safety profile of COVID-19 vaccines.

**Supplementary Information:**

The online version contains supplementary material available at 10.1186/s12916-021-02059-5.

## Introduction

The first coronavirus disease 2019 (COVID-19) case was reported in December 2019 [1]. As of June 15, 2021, more than 175 million COVID-19 cases, including over 3.8 million deaths, were reported in 221 countries and territories [2]. In response to the COVID-19 pandemic, 102 candidate vaccines on 10 platforms are in clinical development, and 15 vaccines have already been licensed or approved for emergency use [3].

These platforms can be classified either as traditional approaches that have previously resulted in licensed vaccines (e.g., inactivated, recombinant proteins, vectored vaccines), or as approaches that have never before been used for a licensed vaccine (e.g., RNA and DNA vaccines) [4]. Since no vaccine against coronaviruses had ever been licensed for use in humans before [4], the rapid process of research and development and limited follow-up time post-vaccination aroused great public concern about the safety profile of COVID-19 vaccine candidates, especially for new platforms such as RNA vaccines. Common reasons given for not intending to receive these vaccines included “concern about the safety of the vaccine in its development” and “potential side effects” [5]. As mass vaccination has progressed, more occurrences of adverse events following immunization (AEFI) have been reported, especially the rare AEFIs. Demonstrating and summarizing vaccine safety from clinical trials and post-authorization surveillance is critical for public confidence, and for enabling timely, evidence-based policy decisions for population-level use [6].

Current evidence about the safety of COVID-19 vaccines relies mainly on data from phase 1–3 randomized controlled trials and vaccine safety surveillance system in several countries. We found three reviews of the safety of COVID-19 vaccines [7–9], which combined study experimental groups, and did not examine the heterogeneity between vaccine platforms and participant age groups. Here, we conduct a rapid review and meta-analysis to summarize the safety data of COVID-19 vaccine candidates. We aim to comprehensively evaluate the rate of solicited, unsolicited, and serious AEFI in each clinical trial and to estimate the relative risk of AEFI by vaccine platform and participant age group. We also collected post-authorization surveillance data from around the world to look for uncommon and delayed onset reactions. This overview of the safety profile of COVID-19 vaccines will support responses to potential safety issues and inform decision-makers evaluating vaccination strategies around the globe.

## Methods

### Data sources and searches

We conducted a rapid review, adhering to the Preferred Reporting Items for Systematic Reviews and Meta-Analyses standards whenever possible. For the published results of clinical trials, we searched PubMed, Embase, and Web of Science for peer-reviewed articles, and PMC for preprints. We also used various combinations of the search terms “severe acute respiratory syndrome coronavirus 2 (SARS-CoV-2)”, “coronavirus”, “vaccines”, “safety”, “adverse event”, and “side effect” to identify relevant regulatory documents disclosing experimental and surveillance data. In addition, we searched official websites and reports using terms for “COVID-19 vaccine safety monitor/monitoring/surveillance” and the names of countries with COVID-19 vaccine programs to identify their available safety surveillance data. Searches were conducted as of June 12, 2021. Details of the search strategy are presented in Additional file 1: Table S1.

### Study selection

Three researchers (Q.W., X.C., X.B.) assessed eligible studies, conducted data extraction, and cross-checked. We looked for clinical trials and post-authorization reports that examined safety data of COVID-19 candidate vaccines, and included manuscripts published in peer-reviewed journals, preprints, and unpublished data disclosed by regulatory agencies. No restrictions were placed on publication date. We excluded study protocols, media news, commentaries, reviews, case reports, reports of non-human clinical trials, reports among specific populations (such as pregnant and lactating women, cancer patients, and other immunosuppressed persons), and abstracts of congress meetings or conference proceedings. We also excluded interim reports of clinical trials that did not clearly show safety data of specific COVID-19 candidate vaccines selected for further use, and reports on vaccines no longer under clinical evaluation. Post-authorization observational studies with sample sizes less than one thousand were excluded as well.

### Data extraction and quality assessment

Information extracted from qualified studies included basic clinical trial details (e.g., study design, study location, phase, arms), characteristics of subjects (e.g., age group, proportion of subjects with underlying conditions, proportion of subjects seropositive at baseline), vaccine formulations (e.g., antigen content, adjuvant, injection route, vaccination schedule), the number of subjects in the safety dataset, and the rate of AEFI during the follow-up period. If data for the same subjects were presented in multiple publications, these data were only counted once. Due to phase 1 and 2 trials often including multiple differing experimental groups, we focused exclusively on safety data from experimental groups in phase 3 clinical trials. Any discrepancies were resolved by consensus or in consultation with a third researcher. Two researchers (Q.W., X.C.) assessed the methodological quality of studies using the Cochrane risk of bias tool [10]. Disagreements were resolved by consensus. Certainty of evidence was assessed by researchers according to the grading recommendations assessment, development and evaluation (GRADE) framework [11, 12].

### Data synthesis and analysis

For the safety profile of COVID-19 vaccines in clinical trials, the primary outcomes were the proportion of vaccine recipients experiencing at least one AEFI and the rates of selected AEFI of COVID-19 candidate vaccines versus placebos. We specified severe versus mild-to-moderate AEFI in our extraction and analyzed these categories separately. For post-authorization safety data, we examined rates of AEFIs, serious adverse events (SAE), and adverse events of special interest (AESI).

We performed meta-analyses of proportions to estimate the pooled rate of safety outcomes of eligible COVID-19 vaccines (i.e., those both with reports of phase 1–3 trials and still under ongoing clinical evaluation) using different platforms. In addition, we estimated the pooled rate ratio (RR) using the rate of safety outcomes of COVID-19 vaccines in vaccinated groups divided by those in control groups in each study. We synthesized evidence for the following events: local reactions (e.g., injection-site pain, injection-site induration, tenderness, swelling), systemic reactions (e.g., nausea, vomiting, fever, rash, myalgia, arthralgia, headache, fatigue, malaise, diarrhea, cough), unsolicited AEFI by system organ class (SOC), AESI, serious AEFI, medically attended events, and study withdrawal of subjects as a result of AEFI and death. Definitions of the study outcomes and the grading scale of selected AEFI were provided in Additional file 1: Table S2-S3.

We explored the reasons for variations among eligible vaccines and examined whether rate of AEFI varied by vaccine platform, age group of participants and serostatus of participants against SARS-CoV-2 at baseline. For the purposes of stratifying safety data by age group, we defined younger adults as under 65 years of age and elderly as over 65 years of age. If the age group of the clinical trial was not completely consistent with our study, the safety data of the closest age stratification was extracted. We classified all participants in the Ad5 nCoV trials as younger adults, since no stratified analyses by age were performed and the proportion of the participants under 55 years old was reported as 86% [13, 14].

Based on a random-effect meta-analysis model, we used the inverse variance method to estimate pooled rate by platform, and the Clopper-Pearson method to calculate 95% confidence intervals [15, 16]. Heterogeneity tests (chi-squared test) with Higgins’ I^2^ statistics were used to determine the extent of variation between vaccines. Multivariate meta-regression models were used to determine the relative contribution of vaccine platform and age of participants to the rate of AEFI. All meta-analyses were performed using per-protocol data. Small study effects (potentially caused by publication bias) were assessed using funnel plots, and formally tested through the rank correlation test for those meta-analyses including more than 10 studies. All statistical analyses were done using R (version 4.0.2), using the “meta” package to conduct the meta-analysis. For all statistical tests, two tailed P-value less than 0.05 were considered statistically significant.

## Results

### Study characteristics

**Fig. 1:**
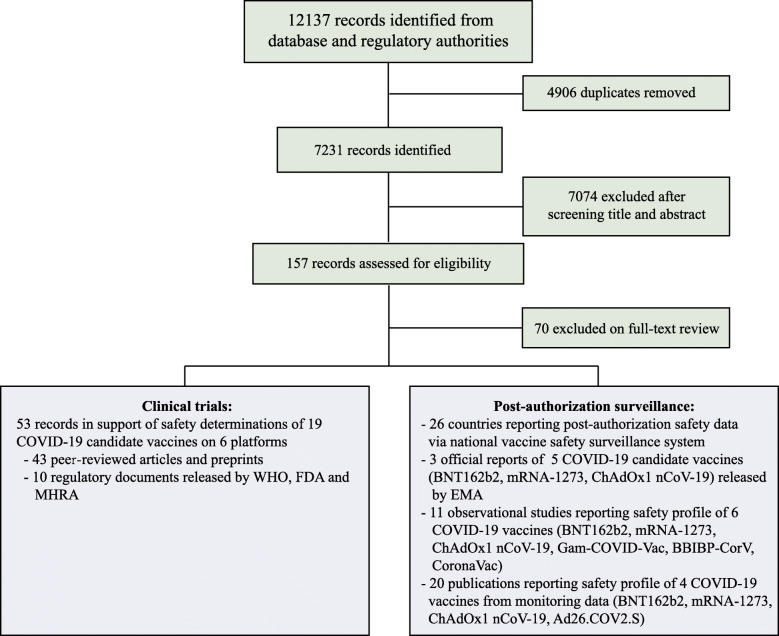
Flow diagram for review process

Our search identified a total of 7231 records after removal of duplicates (Fig. 1). After initial title/abstract screening, 157 articles were assessed for eligibility via full-text review. For the safety data among general population, 43 articles reporting on 19 vaccines of 6 different platforms [14, 17–54] and 10 documents released by WHO (World Health Organization) [55–59], US Food and Drug Administration (FDA) [60–62] and UK Medicines & Healthcare products Regulatory Agency (MHRA) [63, 64] from clinical trials were included. A total of 123,540 study participants receiving COVID-19 vaccines and 97,944 participants receiving placebos were included in safety set of clinical trials. Post-authorization safety profiles were assessed through 3 reports released by the European Medicines Agency (EMA) [65–67], 20 reports including large-scale monitoring data [68–87], 11 observational studies [88–98], and 26 reports from countries’ national surveillance systems.

The main characteristics of included vaccines and relevant clinical trials are reported in Table 1 and Additional file 1: Table S4. The methodological quality of the included studies is reported in Additional file 1: Table S5-S6. Interim and/or final reports of phase 3 clinical trials were available for 8 vaccines: BNT162b2 (RNA vaccine manufactured by Pfizer and BioNTech), mRNA-1273 (RNA vaccine manufactured by Moderna), ChAdOx1-nCoV (non-replicating vector vaccine manufactured by Oxford and AstraZeneca), Gam-COVID-Vac (non-replicating vector vaccine manufactured by Gamaleya Research Institute), Ad26.COV2.S (non-replicating vector vaccine manufactured by Janssen Vaccines & Prevention B.V.), CoronaVac (inactivated vaccine manufactured by SinoVac), BBIBP-CorV and WBIP (inactivated vaccine manufactured by Sinopharm) (Table 1). AEFIs were mainly graded according to the latest scales issued by the US FDA and the China State Food and Drug Administration (CFDA), which are very similar except for a difference of 0.3–0.5 °C in the definition of fever (Table 1 and Additional file 1: Table S3). The funnel plots for safety outcomes including local reaction, systemic reaction, and medically attended events did not appear to be skewed, and the corresponding rank correlation test did not identify asymmetry (Additional file 1: Figure S1).

**Table 1:** Characteristics of included studies reporting safety of COVID-19 candidate vaccines in clinical trials

Platform	Vaccine/manufacturer	Clinical stage	Trial number/study locations	Age range of participants	History of infection	Intervention schedule	Participants included in safety set	Placebo participants	Grading scale
**Inactivated**									
	BBIBP-CorV/Sinopharm	Phase 2/3	ChiCTR2000032459 NCT04510207 ChiCTR2000034780 Global multi-centers	18 years and older	Yes, 6.7% positive at baseline	2 doses, 21 days interval	13,555 (4 μg)	13,481 (aluminum hydroxide)	CFDA, 2019
	WBIP/Sinopharm	Phase 2/3	ChiCTR2000031809 NCT04510207 ChiCTR2000034780 Global	18 years and older	Yes, 6.4% positive at baseline	2 doses, 21 days interval	13,548 (5 μg)	13,481 (aluminum hydroxide)	CFDA, 2019
	CoronaVac/SinoVac	Phase 1/2/3	NCT04352608 NCT04383574 NCT04651790 China, Brazil, Chile	3–17 years old/18–59 years old/60 years and older	No	2 doses, 14/28 days interval	6958 (3 μg)	6629 (aluminum hydroxide)	CFDA, 2019
	IBMCAMS vaccine/Institute of Medical Biology	Phase 1/2	NCT04470609 NCT04412538 China	18–59 years old	No	2 doses, 14 days interval	174 (150 EU)	99 (aluminum hydroxide)	CFDA, 2019
	BBV152 (COVAXIN)/Bharat Biotech	Phase 2	NCT04471519 India	12–65 years old	No	2 doses, 28 days interval	190 (6 μg with Algel-IMDG)	No control groups	FDA and CTCAE
	KCONVAC/Shenzhen Kangtai Biological Products Co., Ltd.	Phase 2	ChiCTR2000038804 ChiCTR2000039462 China	18–59 years old	No	2 doses, 28 days interval	100 (5 μg)	50 (aluminum hydroxide)	CFDA, 2019
**RNA**									
	BNT162b2/Pfizer-BioNTech	Phase 1/2/3	NCT04368728 USA, Argentina, Brazil, Germany, S. Africa, Turkey	12 years and older	Yes	2 doses, 21 days interval	22,752 (30 μg)	22,760 (0.9% sodium chloride)	FDA
	mRNA-1273/Moderna	Phase 3	NCT04283461 USA	18–95 years old	Yes	2 doses, 28 days interval	15,208 (100 μg)	15,210 (0.9% saline)	FDA
	mRNA-1273.351/Moderna	Phase 2	NCT04405076 USA	18 years and older	No	Booster dose	20 (50 μg)	20 (mRNA-1273)	FDA
	CVnCoV/Curevac	Phase 1	NCT04449276 Germany	19–59 years old	No	2 doses, 28 days interval	28 (12 μg)	32 (0.9% saline)	FDA
**Non-replicating viral vector**									
	Ad5 nCoV/CanSino Biological Inc.	Phase 1- 2	NCT04341389 NCT04313127 China	18–83 years old	No	1 dose	165 (5 × 10^10^ vp)	126 (vaccine excipients)	CFDA, 2019
	ChAdOx1-nCoV (AZD1222/Covishield)/AstraZeneca	Phase 1/2/3	NCT04324606 NCT04400838 NCT04444674 ISRCTN 15281137 ISRCTN89951424 Brazil, South Africa, UK	18 years and older	Yes, 3.0% positive at baseline	2 doses, 28 days interval	12,021 (5 × 10^10^ vp or 2.2 × 10^10^ vp)	11,724 (MenACWY^†^ plus saline)	FDA
	Gam-COVID-Vac (Sputnik V)/Gamaleya Research Institute	Phase 1/2/3	NCT04436471 NCT04437875 NCT04530396 Russia	18 years and older	No	2 doses, 21 days interval	16,427 (10^11^ vp for rAd26-S and rAd5-S)	5435 (vaccine buffer composition)	FDA, CTCAE
	Ad26.COV2.S/Johnson & Johnson	Phase 1/2a/3	NCT04436276 NCT04505722 Belgium, US, Argentina, Brazil, Chile, Colombia, Mexico, Peru, South Africa	18–83 years old	Yes, 1.2% positive at baseline	1 dose	21,895 (5 × 10^10^ vp)	21,888	FDA
**Protein subunit**									
	NVX-CoV2373/Novavax	Phase 1–2	NCT04368988 USA, Australia	18–84 years old	No	2 doses, 21 days interval	257 (5μg + 50 μg Matrix-M1)	255 (0.9% saline)	FDA
	SCB-2019/Clover Biopharmaceuticals Inc.	Phase 1	NCT04405908 Australia	18–74 years old	No	2 doses, 21 days interval	16 (30 μg SCB-2019 + CpG/Alum)	30 (0.9% saline)	FDA
	ZF2001/Anhui Zhifei Longcom Biopharmaceutical	Phase 1/2	NCT04445194 NCT04466085 China	20–59 years old	No	3 doses, 30 days interval	170 (25 μg)	160 (aluminum hydroxide)	CFDA, 2019
	EpiVacCorona/Federal Budgetary Research Institution State Research Center of Virology and Biotechnology "Vector"	Phase 1/2	NCT04527575 Russia	18–60 years old	No	2 doses, 21 days interval	57 (225 ± 45 μg)	43 (0.9% saline)	NA
**Virus-like particle**									
	CoVLP/Medicago Inc.	Phase 1	NCT04450004 Canada	19–49 years old	No	2 doses, 21 days interval	20 (3.75 μg + AS03)	No control group	FDA
**DNA**									
	INO-4800/Inovio Pharmaceuticals	Phase 1–2	NCT04336410 NCT04642638 USA	18–80 years old	No	2 doses, 28 days interval	167 (2.0 mg)	50	FDA, CTCAE

*MenACWY* meningococcal group A, C, W, and Y conjugate vaccine; *vp* viral particles; *CTCAE* Common Terminology Criteria for Adverse Events

### Local and systemic reactions in clinical trials

**Fig. 2:**
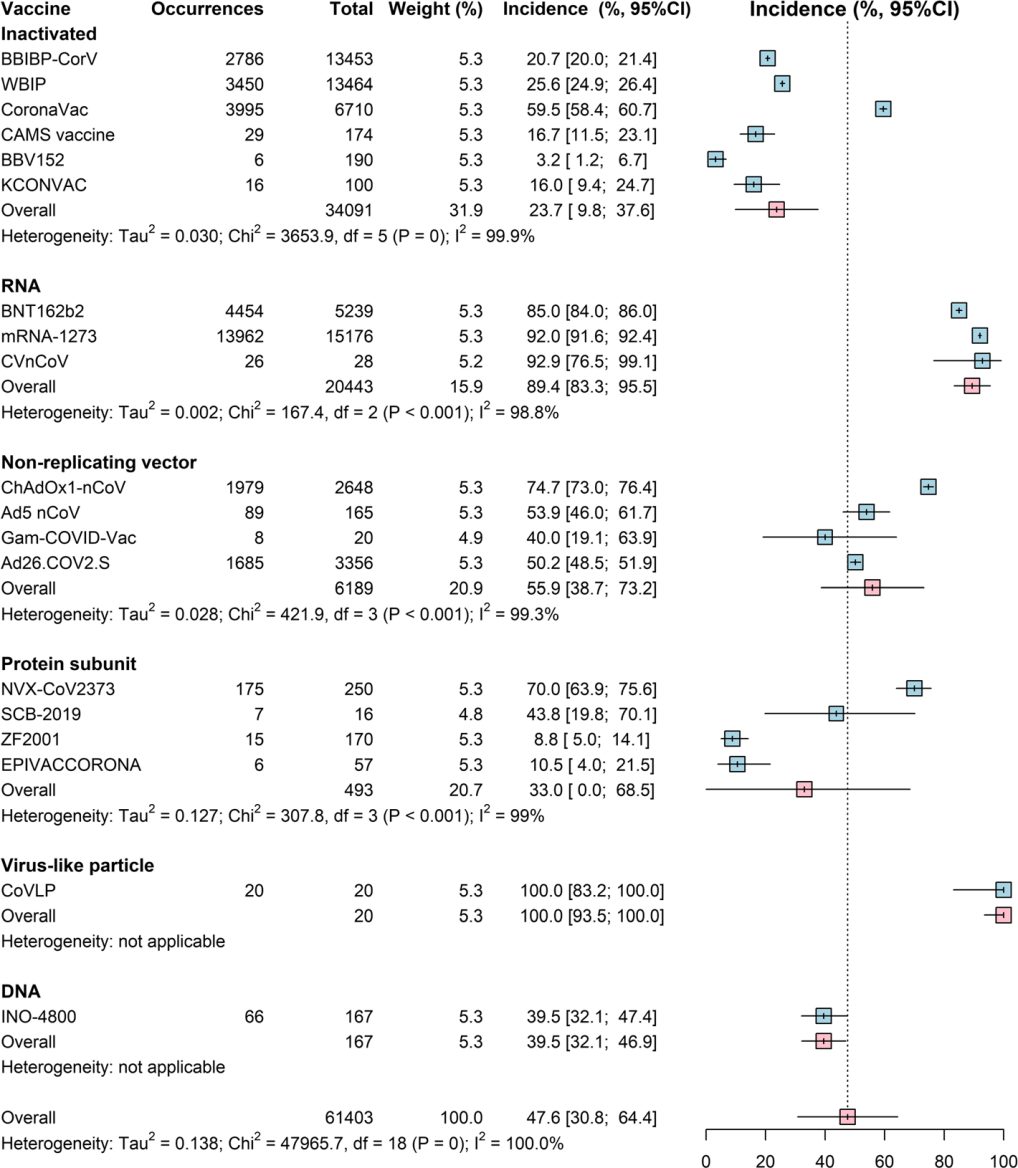
Forest plot of estimated results from meta-analysis of systemic reaction by vaccine platform. The size of the boxes represents the weight for each intervention group. The whisker represents the 95% confidence interval

**Fig. 3:**
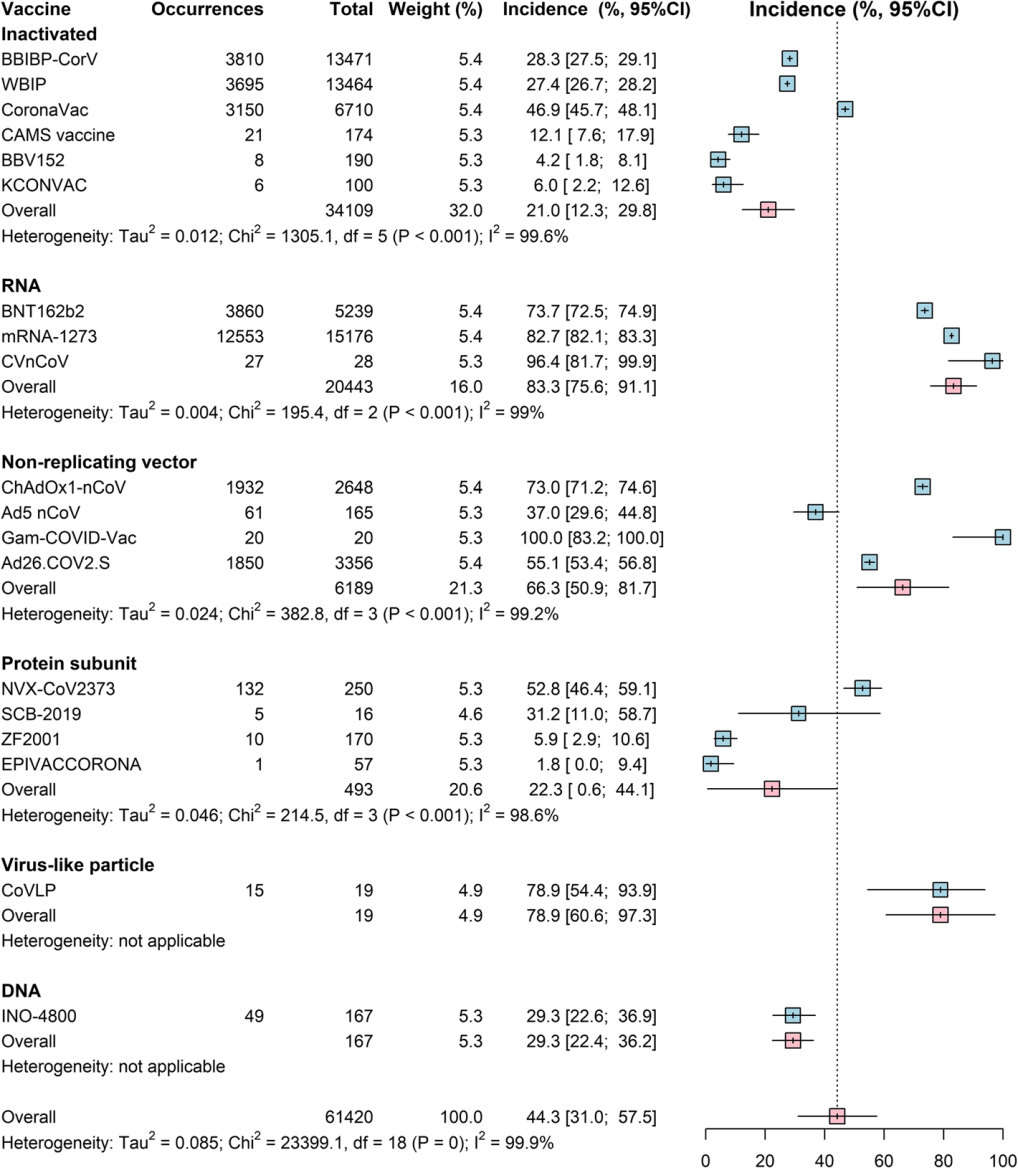
Forest plot of estimated results from meta-analysis of local reaction by vaccine platform. The size of the boxes represents the weight for each intervention group. The whisker represents the 95% confidence interval

The pooled rates of local and systemic reactions, respectively, were significantly lower among inactivated vaccines (23.7%, 21.0%), protein subunit vaccine (33.0%, 22.3%), and DNA vaccines (39.5%, 29.3%) than the 3 other types of COVID-19 vaccines (RNA vaccines, 89.4%, 83.3%; non-replicating vector vaccines, 55.9%, 66.3%; virus-like particle vaccines, 100%, 78.9%) (Figs. 2 and 3). Among all vaccines, solicited injection-site pain and tenderness were the most common local reactions, and fatigue and headache were the most common systemic reactions (Additional file 1: Table S7). Compared to controls, the highest risk of local reactions (RR 4.5, 95% Cl 3.4–5.9) was observed for protein subunit vaccines (Table 2), and a higher risk of medically attended events (RR 1.7, 95% Cl 1.3–2.2) was observed for RNA vaccines (Table 2).

**Table 2:** Summary of findings for safety outcomes in clinical trials

Treatment comparison (reference: placebo)	Study group (N/total)		Pooled RR (95%CI)
	Treatment	Control	
**Local reaction** ***(16 vaccines on 5 platforms)***			
Inactivated vaccines	10,276/33,901	7674/20,033	0.9 (0.8–1.1)
RNA vaccines	18,442/20,443	5393/20,428	**4.0 (2.9–5.4)**
Non-replicating vector vaccines	3753/6169	1926/6003	**2.6 (1.6–4.4)**
Protein subunit vaccines	203/493	45/485	**4.5 (3.4–5.9)**
DNA vaccines	66/167	11/50	**1.8 (1.0–3.1)**
**Systemic reaction** ***(16 vaccines on 5 platforms)***			
Inactivated vaccines	10,682/33,919	6764/20,033	1.0 (1.0–1.0)
RNA vaccines	16,440/20,443	10,505/20,429	**1.6 (1.5–1.6)**
Non-replicating vector vaccines	3843/6169	2694/6003	**1.5 (1.2–1.9)**
Protein subunit vaccines	148/493	105/485	**1.4 (1.2–1.8)**
DNA vaccines	49/167	19/50	0.8 (0.5–1.2)
Medically attended events* ***(2 vaccines on 2 platformss)***			
RNA vaccines	140/15,185	83/15,166	**1.7 (1.3–2.2)**
Non-replicating vector vaccines	304/21,895	408/21,888	0.7 (0.6–0.9)
SAE* ***(8 vaccines on 3 platforms)***			
Inactivated vaccines	156/33,137	109/19,647	0.8 (0.7–1.0)
RNA vaccines	223/37,937	201/37,926	1.1 (0.9–1.3)
Non-replicating vector vaccines	207/50,343	208/39,047	0.8 (0.7–1.0)
SAE related to vaccination* ***(8 vaccines on 3 platforms)***			
Inactivated vaccines	2/33,137	0/19,647	5.0 (0.2–104.0)
RNA vaccines	10/37,937	4/37,926	2.3 (0.5–10.6)
Non-replicating vector vaccines	10/50,343	8/39,047	2.4 (0.7–7.8)

*AEFI* adverse event following immunization, *RR* random-effect risk ratio, *CI* confidence intervals, *N* total number of subjects experiencing one or more AEFI. Per-protocol analysis

*Only considering AEFIs in phase 3 trials

### Unsolicited AEFI, serious AEFI, and AESI in phase 3 clinical trials

For RNA and non-replicating vector vaccines, most unsolicited AEFI and highest risk of unsolicited AEFI by SOC within 28 days post-vaccination were general disorders and administration site conditions, and the rate of common AEFI by SOC was significantly different among vaccines (Additional file 1: Figure S2-S3). The most common serious AEFI by SOC was infections and infestations, while the rate of identified serious AEFIs was similar in the overall vaccine and placebo groups (Additional file 1: Table S8).

For vaccine-related serious AEFI, there was no difference between vaccine and placebo groups (Table 2 and Additional file 1: Table S9). For adverse events of special interest (AESI), approximately 1% and 0.6% of participants vaccinated with RNA vaccines reported hypersensitivity and lymphadenopathy, respectively, and potential risk of hypersensitivity and lymphadenopathy was observed in RNA vaccines compared to control groups (Additional file 1: Table S10). It was worth noting that a total of 7 cases of Bell’s palsy were identified among 36,805 RNA vaccine recipients, indicating a numerical imbalance compared to placebo (Additional file 1: Table S10). There was no imbalance in the number of reported SAEs or grade 3 and over adverse events between vaccine and placebo groups for CoronaVac, BBIBP-CorV, and WBIP.

### Age subgroup analysis based on data from clinical trials

The rate of the most common solicited symptoms was significantly higher among younger adults compared to the elderly (Additional file 1: Table S11). RNA vaccines had significantly higher rate of most common solicited reactions (e.g., injection-site pain, fatigue, headache) among younger adults compared to the other 5 platforms, regardless of the grades of adverse reactions (except overall injection-site pain which was also quite high for virus-like particle vaccines) (Additional file 1: Figures S4-S6). Meanwhile, the highest risk of these common systemic reactions (including fever) was observed in RNA vaccine recipients in this age group, compared to controls (Additional file 1: Table S12). While the highest rate of fever was shown in virus-like particle vaccines (Additional file 1: Figure S7). Differences between vaccine platforms and age groups of vaccine recipients accounted for much of the heterogeneity in safety profiles between COVID-19 vaccines (Additional file 1: Table S13). In addition, the rate of AEFI after CoronaVac was less frequent in children and adolescents than in younger adults, whereas the reverse was found with BNT162b2 (Additional file 1: Table S7).

### Post-authorization observational studies

The most common AEFIs observed in post-authorization observational studies were local injection pain, fatigue, and headache (Additional file 1: Table S14). Adverse events were more frequent in females and subjects with a history of SARS-CoV-2 infection, and decreased with age (Additional file 1: Table S14). Several studies explored COVID-19 vaccination safety signals, including anaphylaxis, cerebral venous sinus thrombosis (CVST), thrombocytopenia, myocarditis, and pericarditis.

### Post-authorization national safety surveillance

Nationwide safety surveillance data for COVID-19 vaccines (mainly BNT162b2, mRNA-1273, ChAdOx1, and nCoV-19) were reported in 26 countries (Additional file 1: Table S15). Most of this reporting was based on passive surveillance and thus not necessarily indicative of true rates or causal relationships with vaccination. Crude reporting rates of common AEFI and SAE varied between countries and were lower than that in clinical trials (Table 3, Additional file 1: Table S16). National rates of anaphylaxis ranged from 2.5 to 15.8 per million doses after mRNA COVID-19 vaccination and were estimated at 0.8 per million doses after Sinopharm vaccination and < 0.5 per million doses after Janssen vaccination (Additional file 1: Table S16).

**Table 3:** Estimated reporting rates of adverse events following immunization (AEFI) from nationwide surveillance by vaccine (per million dose)

Vaccine	Country	Cut-off date	Doses administrated	AEFIs	Crude rate	Pooled rate (95% CI)
**Pfizer/BioNTech**						**3424.5 (2725.7–4123.3)**
	Austria	May 28, 2021	3,495,168	7210	2062.8	
	Belgium	June 8, 2021	3,216,657	8496	2641.3	
	Canada	June 4, 2021	18,894,651	3763	199.2	
	Denmark	June 8, 2021	2,094,751	15,537	7417.1	
	Estonia	June 14, 2021	533,863	1333	2496.9	
	Finland	June 9, 2021	2,587,708	1533	592.4	
	France	June 3, 2021	29,685,000	23,947	806.7	
	Germany	May 31, 2021	36,865,276	34,735	942.2	
	Iceland	June 15, 2021	109,919	624	5676.9	
	Italy	May 26, 2021	22,285,723	47,631	2137.3	
	Netherlands	June 6, 2021	7,300,000	111,852	15,322.2	
	Norway	June 8, 2021	2,414,340	3302	1367.7	
	Spain	March 21, 2021	4,834,876	23,084	4774.5	
	Sweden	June 10, 2021	4,568,479	15,789	3456.1	
	UK	June 2, 2021	25,400,000	193,768	7628.7	
	USA	February 16, 2021	28,374,410	48,196	1698.6	
	Portugal	May 30, 2021	3,943,979	4782	1212.5	
	Slovakia	June 10, 2021	1,961,407	2493	1271.0	
**Moderna**						**8231.3 (7530.6–8931.9)**
	Austria	May 28, 2021	507,987	1645	3238.3	
	Belgium	June 8, 2021	437,008	1787	4089.2	
	Canada	June 4, 2021	5,096,282	2151	422.1	
	Denmark	June 8, 2021	185,169	2510	13,555.2	
	Estonia	June 14, 2021	75,581	159	2103.7	
	Finland	June 9, 2021	298,480	82	274.7	
	France	June 3, 2021	3,492,000	3540	1013.7	
	Germany	May 31, 2021	3,972,764	8319	2094.0	
	Iceland	June 15, 2021	18,502	296	15,998.3	
	Italy	May 26, 2021	2,901,137	2564	883.8	
	Netherlands	June 6, 2021	300,000	20,799	69,330.0	
	Norway	June 8, 2021	318,193	497	1561.9	
	Portugal	May 30, 2021	521,683	387	741.8	
	Slovakia	June 10, 2021	296,050	559	1888.2	
	Spain	March 21, 2021	304,715	2741	8995.3	
	Sweden	June 10, 2021	597,293	4475	7492.1	
	UK	June 2, 2021	460,000	9243	20,093.5	
	USA	February 16, 2021	26,738,383	56,567	2115.6	
**Janssen**						**2683.4 (2070.4–3296.4)**
	Austria	May 28, 2021	36,004	98	2721.9	
	Belgium	June 8, 2021	94,285	167	1771.2	
	Denmark	June 8, 2021	14,019	41	2924.6	
	Estonia	June 14, 2021	16,475	82	4977.2	
	France	June 3, 2021	336,038	80	238.1	
	Germany	May 31, 2021	472,941	733	1549.9	
	Iceland	June 15, 2021	35,726	125	3498.9	
	Italy	May 26, 2021	503,155	171	339.9	
	Netherlands	June 6, 2021	9,000	606	67,333.3	
	Portugal	May 30, 2021	109,409	17	155.4	
	USA	May 7, 2021	7,980,000	13,725	1719.9	
**Oxford/AstraZeneca**						**13,996.5 (10,775.9–17,217.1)**
	Argentina	April 9, 2021	783,055	2069	2642.2	
	Austria	May 28, 2021	941,745	17,132	18,191.8	
	Belgium	June 8, 2021	1,348,696	7078	5248.0	
	Canada	June 4, 2021	2,346,032	874	372.5	
	Denmark	June 8, 2021	150,694	23.236	154.2	
	Estonia	June 14, 2021	203,897	2486	12,192.4	
	Finland	June 9, 2021	406,100	855	2105.4	
	France	June 3, 2021	5,318,878	17,727	3332.8	
	Germany	May 31, 2021	9,230,103	34,870	3777.9	
	Iceland	June 15, 2021	60,044	604	10,059.3	
	Italy	May 26, 2021	6,739,596	15,878	2355.9	
	Netherlands	June 6, 2021	1,300,000	145,423	111,863.8	
	Norway	June 8, 2021	261,624	6640	25,379.9	
	Portugal	May 30, 2021	1,215,009	1509	1242.0	
	Slovakia	June 10, 2021	641,528	2706	4218.1	
	Spain	March 21, 2021	985,528	6343	6436.1	
	Sweden	June 10, 2021	886,815	21,891	24,685.0	
	UK	June 2, 2021	40,200,000	717,250	17,842.0	
**Sinopharm**						**316.4 (285.8–347.0)**
	Argentina	April 9, 2021	1,295,940	410	316.4	
**Sputnik V**						**7447.2 (7356.0–7538.4)**
	Argentina	April 9, 2021	3,414,158	25,426	7447.2	

## Discussion

The pooled rates of local and systemic reactions were significantly different between vaccine platforms. Inactivated vaccines, protein subunit vaccines, and DNA vaccines had lower rates of local and systemic reactions compared to RNA vaccines, non-replicating vector vaccines, and virus-like particle vaccines. The safety profiles of BNT162b2, mRNA-1273, ChAdOx1-nCoV, Ad26.COV2.S, and CoronaVac were relatively benign in the elderly, and both the frequency and the intensity of local and systemic reactions decreased with age. The rates of SAE, including non-fatal serious AEFI and death, were similar in vaccine and placebo groups in clinical trials. Reporting rates of common AEFI after mass public vaccination were lower than in clinical trials. Several unexpected rare adverse events, which resulted in severe outcomes, have been noted in post-authorization surveillance.

Differences in safety profiles of vaccines must be considered in the context of efficacy. Both RNA vaccines (BNT162b2 and mRNA-1273) reported 95% [28] and 94% [99] vaccine efficacy, respectively (symptomatic PCR-confirmed cases were the primary clinical trial outcomes). This is substantially higher than the reported efficacy of other vaccine platforms. The efficacy of inactivated vaccines was reported as 78.1% for BBIBP-CorV [55] and 50.7% for CoronaVac [21]. Efficacy of Ad26.COV2.S against moderate to severe critical Covid-19 with onset at least 14 days after administration was 66.9% [37]. Overall efficacy of ChAdOx1-nCoV in preventing symptomatic COVID-19 across both the low dose and standard dose groups was reported as 70.4% [43]. The efficacy of Gam-COVID-Vac, another non-replicating vector vaccine, was 91.6% [45]. Based on the current evidence, RNA vaccines have both higher rates of adverse reactions and higher efficacy. Due to the relative mild and transient nature of most of these reactions, RNA vaccines should be considered an excellent option to protect against COVID-19, especially in the absence of other viable candidates with similar efficacy. In addition to safety and efficacy, vaccine candidates must also be assessed in the context of the risk of disease, to determine whether each vaccine supports a favorable benefit-risk ratio or not. Such a determination is undoubtedly more important than comparing safety and efficacy between vaccine candidates as long as vaccine supply is limited and disease is prevalent.

Direct comparisons between efficacy data should also be interpreted with caution due to the inconsistency of environmental risk, endpoints, and statistical methods between studies. Current efficacy data show that all authorized vaccines exceed the 50% threshold set by WHO [100], indicating they prevent substantial disease, especially severe cases. Authorized COVID-19 vaccines can prevent a large proportion of symptomatic cases, hospitalizations, severe diseases, and death [101, 102]. Mass vaccination efforts can prevent disease, save lives, reduce pressure on the medical system, and hopefully eventually relieve the need for many of the non-pharmaceutical interventions currently used to contain the epidemic, reopen economies, and allow a return to normalcy worldwide.

As of May 9, 2021, about 0.6 billion people around the world had been vaccinated with at least one dose of COVID-19 vaccines, accounting for about 7.8% of the world’s population [103]. This mass vaccination should allow for the identification of more uncommon and rare AEFI. According to the Vaccine Adverse Event Reporting System (VAERS) and V-safe system of the US Centers for Disease Control and Prevention (CDC) [104], the rates of non-serious AEFI after public administration of BNT162b2 and mRNA-1273 were similar to the clinical trials. Anaphylaxis, a severe, life-threatening allergic reaction, typically occurs at a rate of approximately 1 case per million doses for most vaccines [105]; the rates of anaphylaxis associated with BNT162b2 and mRNA-1273 appear to be approximately 4.7 times and 2.5 times higher than this, respectively, although no cases progressed to serious long-term outcomes thanks to their prompt treatment [106]. Variations in the incidence of anaphylaxis between countries are to be expected, as the numbers vaccinated in most countries to date are relatively small compared with the USA, and the reporting rates of AEFI from passive surveillance are biased. A causal link of thrombosis and thrombocytopenia with adenoviral vector vaccines (ChAdOx1 nCoV-19 and Ad26.COV2.S) was noted after mass public vaccination, including several deaths and severe outcomes [107–110]. While rare side effects should not derail vaccination efforts [111], a thorough risk-benefit analysis is required. Several studies have explored the safety profile of two mRNA vaccines (BNT162b2 and mRNA-1273) in HIV-positive populations [112, 113], immunosuppressive patients [114, 115], and pregnant women [116], revealing no evidence of unexpected serious adverse events. Further evaluation of the benefit-risk profile is warranted in these specific populations.

According to the Chinese government [117], 333 million doses have been administrated as of May 10, 2021 (mainly with BBIBP-CorV and CoronaVac), and the rate of overall AEFI was close to the previous inactivated vaccines given routinely, while the rate of allergic reactions and other non-fatal serious AEFI was about 2 cases per million doses [21]. No major safety concerns have been identified so far. Safety data on Russian vaccines need to be disclosed further so that safety signals can be identified and appropriate risk minimization measures quickly implemented.

The safety profiles of COVID-19 vaccines are still incomplete, even for those currently in use. The safety and efficacy of COVID-19 vaccines in certain subpopulations, such as children and adolescents, pregnant woman, and people with multiple underlying conditions, have not yet been fully studied. Although crude reporting rates of AEFIs from post-authorization safety monitoring have so far been lower than in clinical trials, adverse reactions that are uncommon or have delayed onset require extended post-authorization study to detect. Investigation of safety signals, a lack of epidemiological tools for active surveillance, obstacles at the national regulatory authority level, and a lack of information sharing between countries are still major challenges for most countries. Pharmacovigilance mechanisms must be put in place, with all the necessary training, especially in low- and middle-income countries [118]. Further study will strengthen and expand upon our knowledge in these areas and enable the refinement of vaccine recommendations and injury compensation programs. Safety issues noted in mass vaccination may have a deleterious impact on the global vaccine supply and the already fragile confidence in vaccines. The benefits of vaccines still outweigh the risks at present. Government agencies and vaccine developers should continue to take action to encourage vaccination and reduce public vaccine hesitancy.

Our analysis has several limitations. Firstly, we only included data reported at the study level, due to limited access to individual-level data. Secondly, there are factors we did not include in the meta-analysis, such as seropositivity against SARS-CoV-2 at baseline and underlying conditions, so the potential effects of such heterogeneity were not quantitatively assessed. Thirdly, in the clinical trials for BNT162b2 and ChAdOx1-nCoV, age groups were divided at 55 years of age, which differed from our subgroup analysis of age divided at 65 years of age. Finally, although we included currently available post-authorization safety monitoring data, such monitoring programs are still in their infancy and often rely on a mix of active and passive surveillance.

## Conclusions

In conclusion, the available evidence indicates that eligible COVID-19 vaccines have an acceptable short-term safety profile. Additional studies and long-term population-level surveillance are strongly encouraged to further augment the safety profile of COVID-19 vaccines. This should include essential active vaccine safety surveillance systems, enhanced monitoring of early COVID-19 vaccine recipients and passive surveillance, standardized reporting and pharmacovigilance mechanisms, platforms in hospitals to evaluate the vaccine-specific antibody correlates, and cross-reactivity to other strains. All reports of suspected adverse reactions should be investigated and warning signals rapidly evaluated, to allow implementation of appropriate risk minimization measures and update the benefit/risk ratio of vaccination.

## Supplementary Information

**Additional file 1: Table S1**. Search strategy. **Table S2**. Definitions of outcomes. **Table S3**. Grading scale for selected clinical abnormalities. **Table S4**. Brief description of included COVID-19 candidate vaccines and platforms. **Table S5**. Methodological characteristics of included studies of clinical trials: risk of bias on specific items. **Table S6**. Methodological characteristics of included studies of post-marketing studies: methodological index for non-randomized studies (MINORS) score. **Table S7**. Raw data of common AEFIs in the total safety set for candidate vaccines in clinical trials among general population (n/N, %). **Table S8**. Serious adverse events of COVID-19 vaccines by system organ class in phase 3 clinical trials (n/N, %). **Table S9**. Serious safety outcomes of vaccines in phase 3 clinical trials. **Table S10**. Summary of unbalanced AESIs between intervention and control groups in phase 3 clinical trials of mRNA vaccines. **Table S11**. Age group comparison of most common adverse reactions and fever within 7 days post-vaccination between younger adults and elderly (n/N, %). **Table S12**. Meta-analyses for comparing the rates of most common AEFI of COVID-19 candidate vaccines versus placebo or control vaccine by platform among younger adults (18-65 years old). **Table S13**. Multivariate meta-regression determining factors accounting for the heterogeneity of safety profile. **Table S14**. Summary of post-authorization active surveillance studies among general population. **Table S15**. Sources of nationwide safety surveillance data. **Table S16** Summary of COVID-19 vaccine safety surveillance data. **Figure S1**. Funnel plots to assess publication bias. **Figure S2**. Forest plot of estimated results from meta-analysis of unsolicited adverse events by common system organ class (SOC). **Figure S3**. Comparing rates of unsolicited adverse events by common system organ class (SOC) of COVID-19 vaccines versus placebos. **Figure S4**. Forest plot of estimated results from meta-analysis of local injection pain in adults from clinical trials. **Figure S5**. Forest plot of estimated results from meta-analysis of fatigue in adults from clinical trials. **Figure S6**. Forest plot of estimated results from meta-analysis of headache in adults from clinical trials. **Figure S7**. Forest plot of estimated results from meta-analysis of fever in adults from clinical trials.

## Data Availability

The datasets used and analyzed during the current study are available in appendix.

## Abbreviations

AEFI: Adverse events following immunization
AESI: Adverse events of special interest
CDC: Centers for Disease Control and Prevention
CFDA: China State Food and Drug Administration
COVID-19: Coronavirus diseases 2019
CVST: Cerebral venous sinus thrombosis
EMA: European Medicines Agency
GRADE: Grading Recommendations Assessment, Development and Evaluation
RR: Rate ratio
SAE: Serious adverse events
SARS-CoV-2: Severe acute respiratory syndrome coronavirus 2
SOC: System organ class
UK MHRA: UK Medicines & Healthcare products Regulatory Agency
US FDA: US Food and Drug Administration
VAERS: Vaccine Adverse Event Reporting System
WHO: World Health Organization
