# Supplementary material for: Evaluation of the safety profile of COVID-19 vaccines: a rapid review
Source: BMC Med. 2021 Jul 28;19:173. doi: 10.1186/s12916-021-02059-5 (PMC8315897; doi:10.1186/s12916-021-02059-5)
Supplement: Supplementary file 1 — Additional file 1: Table S1. Search strategy. Table S2. Definitions of outcomes. Table S3. Grading scale for selected clinical abnormalities. Table S4. Brief description of included COVID-19 candidate vaccines and platforms. Table S5. Methodological characteristics of included studies of clinical trials: risk of bias on specific items. Table S6. Methodological characteristics of included studies of post-marketing studies: methodological index for non-randomized studies (MINORS) score. Table S7. Raw data of common AEFIs in the total safety set for candidate vaccines in clinical trials among general population (n/N, %). Table S8. Serious adverse events of COVID-19 vaccines by system organ class in phase 3 clinical trials (n/N, %). Table S9. Serious safety outcomes of vaccines in phase 3 clinical trials. Table S10. Summary of unbalanced AESIs between intervention and control groups in phase 3 clinical trials of mRNA vaccines. Table S11. Age group comparison of most common adverse reactions and fever within 7 days post-vaccination between younger adults and elderly (n/N, %). Table S12. Meta-analyses for comparing the rates of most common AEFI of COVID-19 candidate vaccines versus placebo or control vaccine by platform among younger adults (18-65 years old). Table S13. Multivariate meta-regression determining factors accounting for the heterogeneity of safety profile. Table S14. Summary of post-authorization active surveillance studies among general population. Table S15. Sources of nationwide safety surveillance data. Table S16 Summary of COVID-19 vaccine safety surveillance data. Figure S1. Funnel plots to assess publication bias. Figure S2. Forest plot of estimated results from meta-analysis of unsolicited adverse events by common system organ class (SOC). Figure S3. Comparing rates of unsolicited adverse events by common system organ class (SOC) of COVID-19 vaccines versus placebos. Figure S4. Forest plot of estimated results from meta-analysis of local injection pai [file 12916_2021_2059_MOESM1_ESM.docx]

**Table of Contents**

[Table S1. Search strategy 3](#_Toc76680886)

[Table S2. Definitions of outcomes 10](#_Toc76680887)

[Table S3. Grading scale for selected clinical abnormalities 11](#_Toc76680888)

[Table S4. Brief description of included COVID-19 candidate vaccines and platforms 13](#_Toc76680889)

[Table S5. Methodological characteristics of included studies of clinical trials: risk of bias on specific items 18](#_Toc76680890)

[Table S6. Methodological characteristics of included studies of post-marketing studies: methodological index for non-randomized studies (MINORS) score 21](#_Toc76680891)

[Table S7. Raw data of common AEFIs in the total safety set for candidate vaccines in clinical trials among general population (n/N, %) 24](#_Toc76680892)

[Table S8. Serious adverse events of COVID-19 vaccines by system organ class in phase 3 clinical trials (n/N, %) 32](#_Toc76680893)

[Table S9. Serious safety outcomes of vaccines in phase 3 clinical trials 34](#_Toc76680894)

[Table S10. Summary of unbalanced AESIs between intervention and control groups in phase 3 clinical trials of mRNA vaccines 36](#_Toc76680895)

[Table S11. Age group comparison of most common adverse reactions and fever within 7 days post-vaccination between younger adults and elderly (n/N, %) 37](#_Toc76680896)

[Table S12. Meta-analyses for comparing the rates of most common AEFI of COVID-19 candidate vaccines versus placebo or control vaccine by platform among younger adults (18-65 years old) 47](#_Toc76680897)

[Table S13. Multivariate meta-regression determining factors accounting for the heterogeneity of safety profile 48](#_Toc76680898)

[Table S14. Summary of post-authorization active surveillance studies among general population 50](#_Toc76680899)

[Table S15. Sources of nationwide safety surveillance data 53](#_Toc76680900)

[Table S16 Summary of COVID-19 vaccine safety surveillance data 56](#_Toc76680901)

[Figure S1. Funnel plots to assess publication bias 63](#_Toc76680902)

[Figure S2. Forest plot of estimated results from meta-analysis of unsolicited adverse events by common system organ class (SOC). 65](#_Toc76680903)

[Figure S3. Comparing rates of unsolicited adverse events by common system organ class (SOC) of COVID-19 vaccines versus placebos. 66](#_Toc76680904)

[Figure S4. Forest plot of estimated results from meta-analysis of local injection pain in adults from clinical trials 67](#_Toc76680905)

[Figure S5. Forest plot of estimated results from meta-analysis of fatigue in adults from clinical trials 68](#_Toc76680906)

[Figure S6. Forest plot of estimated results from meta-analysis of headache in adults from clinical trials 69](#_Toc76680907)

[Figure S7. Forest plot of estimated results from meta-analysis of fever in adults from clinical trials 70](#_Toc76680908)

## Table S1. Search strategy

| **No.** | **Terms** | **N** |
| --- | --- | --- |
| **Pubmed** | | |
| #1 | ("COVID-19"[Mesh] OR "SARS-CoV-2"[Mesh] OR "COVID-19 Vaccines"[Mesh] OR "COVID-19 Serological Testing"[Mesh] OR "COVID-19 Nucleic Acid Testing"[Mesh] OR "SARS-CoV-2 variants" [Supplementary Concept] OR "COVID-19 drug treatment" [Supplementary Concept] OR "COVID-19 serotherapy" [Supplementary Concept] OR "2019-nCoV" OR "2019nCoV" OR "cov 2" OR "Covid-19" OR "sars coronavirus 2" OR "sars cov 2" OR "SARS-CoV-2" OR "severe acute respiratory syndrome coronavirus 2" OR “coronavirus 2” OR “COVID 19” OR “COVID-19” OR “2019 ncov” OR “2019nCoV” OR “corona virus disease 2019” OR “cov2” OR “COVID-19” OR “COVID19” OR “nCov 2019” OR “nCoV” OR “new corona virus” OR “new coronaviruses” OR “novel corona virus” OR “novel coronaviruses” OR “SARS Coronavirus 2” OR “SARS2” OR “SARS-COV-2” OR “Severe Acute Respiratory Syndrome Coronavirus 2”) | 141,643 |
| #2 | “vaccines”[MeSH Terms] OR “vaccination”[MeSH Terms] OR “Immunization”[MeSH Terms] OR “vaccin*”[All Fields] OR "immuniz*"[All Fields] | 541,441 |
| #3 | **#1 AND #2** | **13,408** |
| #4 | COVID-19 Vaccines[MeSH Terms] | 2,972 |
| #5 | (BBIBP-CorV[All Fields] OR WBIP[All Fields] OR Sinopharm[All Fields]) OR (CoronaVac[All Fields] OR SinoVac[All Fields]) OR (BBV152[All Fields] OR Covaxin[All Fields]) OR (KCONVAC[All Fields]) OR (ChAdOx1 nCoV-19[All Fields] OR AZD1222[All Fields] OR ChAdOx1-S[All Fields] OR Oxford-AstraZeneca[All Fields] OR Covishield[All Fields]) OR (Ad5 nCoV[All Fields] OR Cansino[All Fields]) OR (Gam-COVID-Vac[All Fields] OR Sputnik V[All Fields]) OR (Ad26.COV2.S[All Fields] OR Janssen COVID-19 Vaccine[All Fields]) OR (BNT162b2[All Fields] OR Pfizer-BioNTech[All Fields] OR Pfizer/BioNTech[All Fields] OR Comirnaty[All Fields]) OR (mRNA-1273[All Fields] OR Moderna COVID-19 vaccine[All Fields]) OR (CVnCoV[All Fields]) OR (NVX-CoV2373[All Fields] OR Novavax[All Fields]) OR (SCB-2019[All Fields]) OR (ZF2001[All Fields]) OR (EpiVacCorona[All Fields]) OR (CoVLP[All Fields]) OR (INO-4800[All Fields]) | 1,453 |
| **#6** | **#3 OR #4 OR #5** | **14,061** |
| #7 | tolerability[Title/Abstract] OR reactogenicity[Title/Abstract] OR safe*[Title/Abstract] OR side effect[Title/Abstract] OR adverse event[Title/Abstract] OR adverse effect[Title/Abstract] OR adverse reaction[Title/Abstract] OR adverse outcome[Title/Abstract] OR undesirable effect*[Title/Abstract] OR treatment emergent[Title/Abstract] OR toxicity[Title/Abstract] | 1,320,494 |
| #8 | myocarditis or pericarditis | 36,605 |
| #9 | Lymphadenopathy | 25,085 |
| #10 | Thrombocytopenia or thrombosis | 308,829 |
| #11 | Anaphylaxis or anaphylactic shock or anaphylactic reaction | 33,441 |
| #12 | Arthropath* or arthrit* or arthralgia | 291,445 |
| #13 | Asthma* | 202,566 |
| #14 | (“Bell? palsy” or “facial paralys*” or “facial neuropath*”) | 15,080 |
| #15 | (Brachial neuritis or brachial plexus neuritis or neuralgia or brachial plexus neuropath*) | 40,844 |
| #16 | (“Myocardial infarction?” or “heart attack” or stroke or “sudden death”) | 625,345 |
| #17 | (“disseminated neuropathy” or “multiple sclerosis” or “neuromyelitis optica”) | 92,441 |
| #18 | (Polyneuropath* or polyradiculoneuropathy or multiple sclerosis or neuromyelitis optica) | 126,469 |
| #19 | (Complex regional pain or causalgia or reflex sympathetic dystrophy) | 12,575 |
| #20 | (Epileps* or myoclon* or spasm* or convulsion? or seizure?) | 310,600 |
| #21 | (Encephalitis or brain inflammation or encephalomyelitis) | 137,976 |
| #22 | Brain disease? or encephal* | 1,549,348 |
| #23 | (Fibromyalgia? or fibrositis or fibrositides or “myofascial pain syndrome”) | 13,254 |
| #24 | (Frozen shoulder or bursitis or synovitis or synovitides or bursitides or adhesive capsulitis or adhesive capsulitides or periarthritis or periarthritides or shoulder impingement or subacromial impingement) | 25,935 |
| #25 | Demyelinating or encephalomyelitis or “Guillain Barre” or myelitis | 160,795 |
| #26 | (“Polyarteritis nodosa” or “periarteritis nodosa” or “essential polyarteritis” or “necrotizing arteritis”) | 7,396 |
| #27 | “Small fiber neuropath*” | 891 |
| #28 | “Systemic lupus” or “Libman-Sacks” | 73,415 |
| #29 | (Syncope or syncopes or syncopal or fainting or (vasovagal adj (collapse or attack or shock or reaction))) | 30,216 |
| #30 | (Vasculitis or vasculitides or angiitis or angiitides or aortitis or arteritis or phlebitis or “Behcet syndrome” or “Wegener granulomatosis” or thrombophlebitis or papulosis) | 123,878 |
| #31 | **#7 OR #8 OR #9 OR #10 OR #11 OR #12 OR #13 OR #14 OR #15 OR #16 OR #17 OR #18 OR #19 OR #20 OR #21 OR #22 OR #23 OR #24 OR #25 OR #26 OR #27 OR #28 OR #29 OR #30** | **4,356,381** |
| #32 | **#6 AND #31** | **3,018** |
| #33 | **Search date: 2020/1/1-2021/6/12** | **2,903** |
| **Web of Science** | | |
| #1 | TS=(“Wuhan coronavirus” OR “COVID19*” OR “COVID-19*” OR “COVID-2019*” OR “coronavirus disease 2019” OR “SARS-CoV-2” OR “2019-nCoV” OR “2019 novel coronavirus” OR “severe acute respiratory syndrome coronavirus 2” OR “2019 novel coronavirus infection” OR “coronavirus disease 2019” OR “coronavirus disease-19” OR “SARS-CoV-2019” OR “SARS-CoV-19”) | 181,867 |
| #2 | TS = (vaccin* OR immuniz*) | 1,138,844 |
| **#3** | **#1 AND #2** | **15,005** |
| #4 | TS = ((BBIBP-CorV OR WBIP OR Sinopharm) OR (CoronaVac OR SinoVac) OR (BBV152 OR Covaxin) OR (KCONVAC) OR ("ChAdOx1 nCoV-19" OR AZD1222 OR ChAdOx1-S OR Oxford-AstraZeneca OR Covishield) OR ("Ad5 nCoV" OR Cansino) OR (Gam-COVID-Vac OR "Sputnik V") OR (Ad26.COV2.S OR "Janssen COVID-19 Vaccine") OR (BNT162b2 OR Pfizer-BioNTech OR Pfizer/BioNTech OR Comirnaty) OR (mRNA-1273 OR "Moderna COVID-19 vaccine") OR (CVnCoV) OR (NVX-CoV2373 OR Novavax) OR (SCB-2019) OR (ZF2001) OR (EpiVacCorona) OR (CoVLP) OR (INO-4800)) | 918 |
| **#5** | **#3 OR #4** | 15,133 |
| #6 | TS = (tolerability OR reactogenicity OR safe* OR "side effect" OR "adverse event" OR "adverse effect" OR "adverse reaction" OR "adverse outcome" OR "undesirable effect" OR "treatment emergent" OR "toxcity") | 5,107,255 |
| #7 | TS = (myocarditis or pericarditis) | 56,803 |
| #8 | TS = (Lymphadenopathy) | 38,192 |
| #9 | TS = (Thrombocytopenia or thrombosis) | 418,327 |
| #10 | TS = (Anaphylaxis or "anaphylactic shock" or "anaphylactic reaction") | 56,411 |
| #11 | TS = (Arthropath* or arthrit* or arthralgia) | 535,873 |
| #12 | TS = (Asthma*) | 372,196 |
| #13 | TS = (“Bell? palsy” or “facial paralys*” or “facial neuropath*”) | 17,583 |
| #14 | TS = (Brachial neuritis or brachial plexus neuritis or neuralgia or brachial plexus neuropath*) | 48,116 |
| #15 | TS = (“Myocardial infarction?” or “heart attack” or stroke or “sudden death”) | 806,426 |
| #16 | TS = (“disseminated neuropathy” or “multiple sclerosis” or “neuromyelitis optica”) | 209,648 |
| #17 | TS = (Polyneuropath* or polyradiculoneuropathy or multiple sclerosis or neuromyelitis optica) | 255,536 |
| #18 | TS = (Complex regional pain or causalgia or reflex sympathetic dystrophy) | 12,102 |
| #19 | TS = (Epileps* or myoclon* or spasm* or convulsion? or seizure?) | 449,435 |
| #20 | TS = (Encephalitis or brain inflammation or encephalomyelitis) | 225,304 |
| #21 | TS = (Brain disease? or encephal*) | 613,830 |
| #22 | TS = (Fibromyalgia? or fibrositis or fibrositides or “myofascial pain syndrome”) | 3,816 |
| #23 | TS = (Frozen shoulder or bursitis or synovitis or synovitides or bursitides or adhesive capsulitis or adhesive capsulitides or periarthritis or periarthritides or shoulder impingement or subacromial impingement) | **47164** |
| #24 | TS = (Demyelinating or encephalomyelitis or “Guillain Barre” or myelitis) | 128,123 |
| #25 | TS = (“Polyarteritis nodosa” or “periarteritis nodosa” or “essential polyarteritis” or “necrotizing arteritis”) | 12,279 |
| #26 | TS = (“Small fiber neuropath*”) | **1,882** |
| #27 | TS = (“Systemic lupus” or “Libman-Sacks”) | 130,395 |
| #28 | TS = (Syncope or syncopes or syncopal or fainting or (vasovagal adj (collapse or attack or shock or reaction))) | 78,331 |
| #29 | TS = (Vasculitis or vasculitides or angiitis or angiitides or aortitis or arteritis or phlebitis or “Behcet syndrome” or “Wegener granulomatosis” or thrombophlebitis or papulosis) | 160,552 |
| **#30** | **#6 OR #7 OR #8 OR #9 OR #10 OR #11 OR #12 OR #13 OR #14 OR #15 OR #16 OR #17 OR #18 OR #19 OR #20 OR #21 OR #22 OR #23 OR #24 OR #25 OR #26 OR #27 OR #28 OR #29** | **8,505,158** |
| **#31** | **#5 AND #30** | **3,050** |
| **#32** | **Search date: 2020/1/1-2021/6/12** | **3,029** |
| **Embase** | | |
| #1 | ('Severe acute respiratory syndrome coronavirus 2'/exp) OR (‘2019-nCoV’ OR ‘2019nCoV’ OR ‘cov 2’ OR ‘Covid-19’ OR ‘sars coronavirus 2’ OR ‘sars cov 2’ OR ‘SARS-CoV-2’ OR ‘severe acute respiratory syndrome coronavirus 2’ OR ‘coronavirus 2’ OR ‘COVID 19’ OR ‘COVID-19’ OR ‘2019 ncov’ OR ‘2019nCoV’ OR ‘corona virus disease 2019’ OR ‘cov2’ OR ‘COVID-19’ OR ‘COVID19’ OR ‘nCov 2019’ OR ‘nCoV’ OR ‘new corona virus’ OR ‘new coronaviruses’ OR ‘novel corona virus’ OR ‘novel coronaviruses’ OR ‘SARS Coronavirus 2’ OR ‘SARS2’ OR ‘SARS-COV-2’ OR ‘Severe Acute Respiratory Syndrome Coronavirus 2’):ti,ab,kw OR (19 OR 2019 OR ‘2019-nCoV’ OR ‘Beijing’ OR ‘China’ OR ‘Covid-19’ OR epidem* OR epidemic* OR epidemy OR new OR ‘novel’ OR ‘outbreak’ OR pandem* OR ‘SARS-CoV-2’ OR ‘Shanghai’ OR ‘Wuhan’):ti,ab,kw AND ('Coronavirinae'/exp OR 'Coronavirus infection'/exp OR coronavirus*:ti,ab,kw OR corona-virus*:ti,ab,kw OR cov:ti,ab,kw OR ‘pneumonia-virus*’:ti,ab,kw) | 144,270 |
| #2 | vaccine OR vaccine/exp OR vaccination OR vaccination/exp OR immunization OR immunization/exp | 613,560 |
| #3 | **#1 AND #2** | **13,143** |
| #4 | "BBIBP-CorV" OR WBIP OR Sinopharm OR CoronaVac OR SinoVac OR BBV152 OR Covaxin OR KCONVAC OR "ChAdOx1 nCoV-19" OR AZD1222 OR ChAdOx1-S OR Oxford-AstraZeneca OR Covishield OR "Ad5 nCoV" OR Cansino OR Gam-COVID-Vac OR "Sputnik V" OR Ad26.COV2.S OR "Janssen COVID-19 Vaccine" OR BNT162b2 OR Pfizer-BioNTech OR Comirnaty OR mRNA-1273 OR "Moderna COVID-19 vaccine" OR CVnCoV OR NVX-CoV2373 OR Novavax OR SCB-2019 OR ZF2001 OR EpiVacCorona OR CoVLP OR INO-4800 | 2,473 |
| **#5** | **#3 OR #4** | **14,817** |
| #6 | (tolerability OR reactogenicity OR safe* OR "side effect" OR "adverse event" OR "adverse effect" OR "adverse reaction" OR "adverse outcome" OR "undesirable effect" OR "treatment emergent" OR "toxcity"):ti,ab,kw | 1,618,152 |
| #7 | myocarditis or pericarditis | 58,370 |
| #8 | Lymphadenopathy | 76,318 |
| #9 | thrombocytopenia | 170,576 |
| #10 | thrombosis | 448,212 |
| #11 | anaphylaxis OR 'anaphylactic shock' | 62,136 |
| #12 | arthralgia'/exp OR 'arthritis'/exp OR 'arthropathy'/exp | 858,384 |
| #13 | Asthma or "status asthmaticus" | 342,921 |
| #14 | "Bell palsy" | 3,921 |
| #15 | "Brachial plexus neuritis" | 107 |
| #16 | "myocardial infarction"/exp or stroke/exp or "death, sudden"/exp | 752,687 |
| #17 | "Chronic inflammatory demyelinating polyneuropathy" or "polyradiculoneuropathy, chronic inflammatory demyelinating" or "multiple sclerosis"/exp or "neuromyelitis optica" | 149,371 |
| #18 | "complex regional pain syndromes"/exp or "complex regional pain syndrome"/exp | 10,926 |
| #19 | "Seizures" or "seizures, febrile" or "convulsion" or "epilepsies, myoclonic"/exp or "spasms, infantile" or "myoclonus" | 196,595 |
| #20 | "encephalitis"/exp | 116,967 |
| #21 | "brain disease"/exp or encephalopathy*:ti,ab | 2,335,395 |
| #22 | Fibromyalgia | 23,773 |
| #23 | "Frozen shoulder" or "bursitis"/exp or "shoulder impingement syndrome" or "synovitis"/exp | 37,441 |
| #24 | "Guillain-Barre syndrome"/exp | 16,001 |
| #25 | "Polyarteritis nodosa" | 9,571 |
| #26 | “Small fiber neuropath*”:ti,ab | 1,460 |
| #27 | "lupus erythematosus, systemic"/exp | 102,322 |
| #28 | syncope/exp | 49,497 |
| #29 | Vasculitis/exp | 134,151 |
| #30 | **#6 OR #7 OR #8 OR #9 OR #10 OR #11 OR #12 OR #13 OR #14 OR #15 OR #16 OR #17 OR #18 OR #19 OR #20 OR #21 OR #22 OR #23 OR #24 OR #25 OR #26 OR #27 OR #28 OR #29** | **6,024,246** |
| #31 | **#5 AND #30** | **3,120** |
| #32 | **Search date: 2020/1/1-2021/6/12** | **2,516** |
| **PMC** | | |
| #1 | (COVID-19 vaccine) AND (tolerability OR reactogenicity OR safe* OR "side effect" OR "adverse event" OR "adverse effect" OR "adverse reaction" OR "adverse outcome" OR "undesirable effect" OR "treatment emergent" OR "toxicity") AND (SRC:PPR) | 3,676 |

## Table S2. Definitions of outcomes

| **Outcome** | **Definition** |
| --- | --- |
| Adverse event following immunization (AEFI) | An AEFI is any untoward medical occurrence which follows  immunization and which does not necessarily have a causal relationship with the usage of the vaccine. The adverse event may be any unfavorable or unintended sign, abnormal laboratory finding, symptom or disease. |
| Serious adverse events (SAE) | A serious adverse event is any untoward medical occurrence that at any dose results in death, requires inpatient hospitalization or prolongation of existing hospitalization, results in persistent or significant disability/incapacity, or is life-threatening. |
| Solicited adverse events | Solicited adverse events include prospectively self-collected occurrences of local and systemic reactions. Participants were usually asked to monitor and record local reactions, systemic events, and antipyretic medication usage for 7 days following each administration. |
| Local reactions | Local reactions included pain at the injection site, redness, swelling, induration, etc. |
| Systemic reactions | Systemic reactions included headache, myalgia, fever, fatigue, vomiting, diarrhea, muscle pain, etc. |
| Unsolicited adverse events | Unsolicited AEs would be represented in the AE domain unless they were classified as solicited adverse event. |
| Adverse event of special interest (AESI) | An adverse event of special interest (serious or non-serious) is one of scientific and medical concern specific to vaccine, for which ongoing monitoring can be appropriate. |
| Withdrawal due to adverse events | The number of participants reported as withdrawn from clinical trial due to adverse events whether related to study intervention or not. |
| Death | The number of participants reported for death regardless of causality. |

## Table S3. Grading scale for selected clinical abnormalities

| **Criteria for**  **grading scale** | **Grades** | **Injection pain** | **Headache** | **Fatigue** | **Fever (°C) *** |
| --- | --- | --- | --- | --- | --- |
| FDA |  |  |  |  |  |
|  | Mild  (Grade 1) | Does not interfere with activity | No interference with activity | No interference with activity | 38.0 ~ 38.4 (oral temperature) |
|  | Moderate  (Grade 2) | Repeated use of non-narcotic pain reliever > 24 hours or interferes with activity | Repeated use of non-narcotic pain reliever > 24 hours or some interference with activity | Some interference with activity | 38.5 ~ 38.9 (oral temperature) |
|  | Severe  (Grade 3) | Any use of narcotic pain reliever or prevents daily activity | Significant; any use of narcotic pain reliever or prevents daily activity | Significant; prevents daily activity | 39.0 ~ 40 (oral temperature) |
|  | Potentially Life  Threatening  (Grade 4) | ER visit or hospitalization | ER visit or hospitalization | ER visit or hospitalization | > 40 (oral temperature) |
| CFDA |  |  |  |  |  |
|  | Mild  (Grade 1) | Does not interfere with activity | No impact on daily activities, no need for treatment | No impact on daily activities | 37.3 ~ 38.0 (axillary temperature) |
|  | Moderate  (Grade 2) | Interferes with activity | Temporary, slight impact on daily activities, may need treatment or intervention | Affect normal daily activities | 38.0 ~ 38.5 (axillary temperature) |
|  | Severe  (Grade 3) | Prevents daily activity | Seriously affecting daily activities, requiring treatment or intervention | Seriously affect daily activities, unable to work | 38.5 ~ 39.5 (axillary temperature) |
|  | Potentially Life  Threatening  (Grade 4) | Loss of basic self-care ability, or hospitalization | Intractable, requiring emergency treatment or hospitalization | Hospitalization | ≥39.5 (axillary temperature), lasting for more than 3 days |

Note: ER represented emergency room.

*: Oral temperature = axillary temperature+0.2°C, Anal temperature = axillary temperature+(0.3 - 0.5)°C.

## Table S4. Brief description of included COVID-19 candidate vaccines and platforms

| **Platform** | **Vaccine** | **Description** |
| --- | --- | --- |
| Inactivated vaccine | | Consist of virus particles, bacteria, or other pathogens that have been grown in culture and then lose disease producing capacity. |
|  | BBIBP-CorV | BBIBP-CorV was developed by the Beijing Institute of Biological Products (Beijing, China) and manufactured as a liquid formulation containing 4 μg total protein with aluminium hydroxide adjuvant (0·45 mg/ml) per 0·5 ml. β-propionolactone was thoroughly mixed with the harvested viral solution at a ratio of 1:4000 at 2-8°C to inactivate virus production. |
|  | WBIP | WBIP was developed by the Wuhan Institute of Biological Products (Wuhan, China) and adsorbed to 0.5-mg alum and packed into prefilled syringes in 0.5-mL sterile phosphate-buffered saline without preservative. A SARS-CoV-2 strain (WIV04 strain) was inactivated by β-propiolactone twice. |
|  | IBMCAMS vaccine | The SARS-CoV-2 inactivated vaccine was developed by the Institute of Medical Biology (IMB), Chinese Academy of Medical Sciences (CAMS). The vaccine contained 150 EU of inactivated viral antigen adsorbed to 0.25mg of Al(OH)_3_ adjuvant and suspended in 0.5 ml of buffered saline for each dose. |
|  | CoronaVac | CoronaVac was created from African green monkey kidney cells (Vero cells) that have been inoculated with SARS-CoV-2 (CN02 strain). The vaccine was inactivated with β-propiolactone, concentrated, purified, and finally absorbed onto aluminium hydroxide. |
|  | BBV152 (Covaxin) | BBV152 (manufactured by Bharat Biotech) is a whole-virion ß-propiolactone-inactivated SARSCoV-2 vaccine. The vaccine strain NIV-2020-770 contains the D614G mutation, which is characterized by an aspartic acid to glycine shift at amino acid position 614 of the spike protein. The candidates were formulated with Algel-IMDG, an imidazoquinolinone class molecule (a Tolllike receptor (TLR)7/TLR8 agonist abbreviated as IMDG) adsorbed to Algel. |
|  | KCONVAC | KCONVAC was developed and manufactured by Shenzhen Kangtai Biological Products Co., Ltd. (China) and Beijing Minhai Biotechnology Co., Ltd. (China). The vaccine strain of SARS-CoV-2 virus (19nCoV-CDC-Tan-Strain03) was cultivated in Vero cells. The harvested virus was inactivated by treatment with β-propiolactone, purified, and adsorbed to aluminum hydroxide (adjuvant). |
| Non−replicating viral vector vaccine | | Non−replicating viral vector vaccine is a vaccine that uses a replication-defective [virus](https://en.wikipedia.org/wiki/Virus) viral vector (such as) to transport pieces of the pathogen in order to stimulate an [immune response](https://en.wikipedia.org/wiki/Immune_response). Several viruses, including adenovirus, adeno-associated virus, measles virus, and human parainfluenza virus, are widely used as viral vectors. Non-replicating viral vectors do not make extra copies of themselves in our body’s cells. |
|  | ChAdOx1-nCoV  (AZD1222/Covishield) | ChAdOx1 nCoV-19 was manufactured by the Clinical BioManufacturing Facility (University of Oxford, Oxford, UK), which used a replication-deficient simian adenovirus vector ChAdOx1, containing the full-length structural surface glycoprotein (spike protein) of SARS-CoV-2. ChAdOx1 nCoV-19 was administered at a standard dose of 3.5-6.5 × 10¹⁰ viral particles and a low dose of 2.2 × 10¹⁰ viral particles. |
|  | Ad5 nCoV | The Ad5 vectored COVID-19 vaccine was developed by Beijing Institute of Biotechnology (Beijing, China) and CanSino Biologics (Tianjin, China), and manufactured as a liquid formulation containing 5 × 10¹⁰ viral particles per 0.5 ml in a vial.  The vaccine is a replication defective Ad5 vectored vaccine expressing the spike glycoprotein of SARS-CoV-2. |
|  | Gam-COVID-Vac  (Sputnik V) | The vaccine was developed by N F Gamaleya National Research Centre and manufactured as two formulations, frozen and lyophilised. The vaccine comprises two vector components, recombinant adenovirus type 26 (rAd26) and recombinant adenovirus type 5 (rAd5), both of which carry the gene for SARS-CoV-2 full-length glycoprotein S. |
|  | Ad26.COV2.S | The vaccine was a recombinant, replication-incompetent adenovirus serotype 26 (Ad26) vector encoding a full-length and stabilized SARS-CoV-2 spike protein and developed by Janssen Vaccines and Prevention B.V. |
| RNA vaccine | | Consist of messenger RNA molecules which code for parts of the target pathogen that are recognized by our immune system. |
|  | BNT162b2 | The vaccine was a lipid nanoparticle-formulated, nucleoside-modified mRNA which encodes the SARS-CoV-2 full length spike and developed by BioNTech and Pfizer. |
|  | mRNA-1273/  mRNA-1273.351 | The vaccine was a lipid nanoparticle-encapsulated, nucleoside-modified messenger RNA (mRNA)-based vaccine that encodes the SARS-CoV-2 spike(S) glycoprotein and developed by Moderna. The mRNA-1273.351 vaccine, like mRNA-1273, encodes the prefusion stabilized S protein of SARS-CoV-2 with the key amino acid changes present in the B.1.351 strain of the virus. |
|  | CVnCoV | The vaccine contained sequence optimized mRNA coding for a stabilized form of S protein encapsulated in lipid nanoparticles (LNP) and was developed by CureVac AG. |
| Protein subunit vaccine | | Protein vaccines are composed of purified or recombinant proteinaceous antigens from a pathogen, such as a bacterium or virus. |
|  | NVX-CoV2373 | The vaccine was developed by Novavax and manufactured as a blend of 5 μg rSARS-CoV-2 and 50 μg Matrix-M1.  rSARS-CoV-2 is a recombinant nanoparticle vaccine constructed from the full-length, wild-type SARS-CoV-2 spike glycoprotein optimized in the established baculovirus Spodoptera frugiperda (Sf9) insect cell expression system. Matrix-M1 is a saponin-based adjuvant. |
|  | SCB-2019 | The vaccine was a novel protein subunit vaccine candidate composed of a stabilised trimeric form of the spike (S)-protein produced in CHO cells, combined with adjuvant AS03 and developed by Clover Biopharmaceuticals AUS Pty Ltd |
|  | ZF2001 | The vaccine was jointly developed by Institute of Microbiology, Chinese Academy of Sciences and Anhui Zhifei Longcom Biopharmaceutical Co., Ltd. The recombined vaccine encodes the SARS-CoV-2 RBD antigen (residues 319-537) in dimeric form and was manufactured as a liquid formulation containing 25 μg per 0.5 mL in a vial, with aluminum hydroxide as the adjuvant. |
|  | EpiVacCorona | The EpiVacCorona Vaccine was developed and manufactured by FBRI SRC VB VECTOR, Rospotrebnadzor (Russia, Koltsovo). The carrier protein MBP-6xHis-N_nCoV-2019 contains the structures of SARS-CoV-2 N protein, the E. coli maltose binding protein (MBP),  and the 6xHis-tag required for purification with metal chelate affinity chromatography. SARS-CoV-2 N protein was chosen since it is well-conserved and contains virus-specific T-cell epitopes and thus should be also involved in production of memory T cells. After the purification stage, the carrier protein is covered with covalently bound peptides and adsorbed on aluminum hydroxide used as adjuvant. |
| Virus-like particle vaccine | | Virus-like particles (VLPs) are molecules that closely resemble viruses, but are non-infectious because they contain no viral genetic material. They can be naturally occurring or synthesized through the individual expression of viral structural proteins, which can then self-assemble into the virus-like structure. VLPs can be produced in multiple cell culture systems including bacteria, mammalian cell lines, insect cell lines, yeast and plant cells. |
|  | CoVLP | CoVLP is developed by Medicago and produced by transient transfection of Nicotiana benthamiana plants. These VLPs spontaneously assemble at the plant cell membrane and display SARS-COV-2 trimers of stabilized pre-fusion S protein on their surface. The vaccine was administered as a blend of 3.75 μg S protein content and adjuvanted AS03. |
| DNA vaccine | | Consist of DNA molecules which are converted into antigens by our body's cells. |
|  | INO-4800 | INO-4800 was developed by Inovio Pharmaceuticals, containing plasmid pGX9501 expressing a synthetic, optimized sequence of the SARS-CoV-2 full length spike glycoprotein which was optimized at a concentration of 10 mg/ml in a saline sodium citrate buffer. |

## Table S5. Methodological characteristics of included studies of clinical trials: risk of bias on specific items

| **Vaccine** | **Clinical phase** | **Study ID** | **Randomization process** | **Deviations from intended interventions** | **Missing outcome data** | **Measurement of the outcome** | **Selection of the reported result** | **Overall bias** |
| --- | --- | --- | --- | --- | --- | --- | --- | --- |
| ***Inactivated vaccine*** | | | | | | | | |
| BBIBP-CorV | Phase 1/2 | Xia, Lancet Infect Dis 2020 | Low | Low | Low | Low | Low | Low |
| WBIP | Phase 1/2 | Xia, JAMA 2020 | Low | Low | Low | Low | Low | Low |
| BBIBP-CorV/WBIP | Phase 3 | Kaabi, JAMA 2021 | Low | Low | Low | Low | Low | Low |
| CoronaVac | Phase 1/2 | Zhang, Lancet Infect Dis 2020 | Low | Low | Low | Low | Low | Low |
| CoronaVac | Phase 1/2 | Wu, Lancet Infect Dis 2021 | Low | Low | Low | Low | Low | Low |
| CoronaVac | Phase 1/2 | Han, SSRN 2021 | Low | Low | Low | Low | Low | Low |
| CoronaVac | Phase 3 | Bueno, MedRxiv 2021 | Low | Low | Low | Low | Low | Low |
| CoronaVac | Phase 3 | Palacios, SSRN 2021 | Low | Low | Low | Low | Low | Low |
| IBMCAMS vaccine | Phase 1 | Pu, Vaccine 2021 | Low | Low | Low | Low | Low | Low |
| IBMCAMS vaccine | Phase 2 | Che, CID 2020 | Low | Low | Low | Low | Low | Low |
| BBV152 | Phase 1/2 | Ella, Lancet Infect Dis 2021 | Low | Low | Low | Low | Low | Low |
| KCONVAC | Phase 1/2 | Pan, Chinese Medical Journal 2021 | Low | Low | Low | Low | Low | Low |
| ***RNA vaccine*** | | | | | | | | |
| BNT162b2 | Phase 1 | Walsh, NEJM 2020 | Low | Low | Low | Low | Low | Low |
| BNT162b2 | Phase 1/2 | Sahin, MedRxiv 2020 | Some concerns | Low | Low | Low | Low | Some concerns |
| BNT162b2 | Phase 3 | Polack, NEJM 2020 | Low | Low | Low | Low | Low | Low |
| BNT162b2 | Phase 3 | Frenck, NEJM 2021 | Low | Low | Low | Low | Low | Low |
| mRNA-1273 | Phase 1 | Anderson, NEJM 2020 | Some concerns | Low | Low | Low | Low | Some concerns |
| mRNA-1273 | Phase 1 | Jackson, NEJM 2020 | Some concerns | Low | Low | Low | Low | Some concerns |
| mRNA-1273 | Phase 3 | Baden, NEJM 2020 | Low | Low | Low | Low | Low | Low |
| mRNA-1273 | Phase 2 | Chu, Vaccine 2021 | Low | Low | Low | Low | Low | Low |
| mRNA-1273.351 | Phase 2 | Wu, MedRxiv 2021 | Some concerns | Low | Low | Low | Low | Some concerns |
| CVnCoV | Phase 1 | Kremsner, MedRxiv 2020 | Low | Low | Low | Low | Low | Low |
| ***Non-replicating vector vaccine*** | | | | | | | | |
| Ad5 nCoV | Phase 1 | Zhu, Lancet 2020 | Some concerns | Low | Low | Low | Low | Some concerns |
| Ad5 nCoV | Phase 2 | Zhu, Lancet 2020 | Low | Low | Low | Low | Low | Low |
| ChAdOx1-nCoV | Phase 1/2 | Folegatti, Lancet 2020 | Low | Low | Low | Low | Low | Low |
| ChAdOx1-nCoV | Phase 1/2 | Barrett, Nature medicine 2021 | Low | Low | Low | Low | Low | Low |
| ChAdOx1-nCoV | Phase 2 | Ramasamy, Lancet 2020 | Low | Low | Low | Low | Low | Low |
| ChAdOx1-nCoV | Phase 3 | Voysey, Lancet 2020 | Low | Low | Low | Low | Low | Low |
| ChAdOx1-nCoV | Phase 3 | Voysey, Lancet 2021 | Low | Low | Low | Low | Low | Low |
| ChAdOx1-nCoV | Phase 3 | Madhi, NEJM 2021 | Low | Low | Low | Low | Low | Low |
| Gam-COVID-Vac | Phase 1/2 | Logunov, Lancet 2020 | Some concerns | Low | Low | Low | Low | Some concerns |
| Gam-COVID-Vac | Phase 3 | Logunov, Lancet 2021 | Low | Low | Low | Low | Low | Low |
| Ad26.COV2.S | Phase 1/2a | Sadoff, NEJM 2021 | Low | Low | Low | Low | Low | Low |
| Ad26.COV2.S | Phase 3 | Sadoff, NEJM 2021 | Low | Low | Low | Low | Low | Low |
| GRAd-COV2 | Phase 1 | Lanini, MedRxiv 2021 | Some concerns | Low | Low | Low | Low | Some concerns |
| ***Protein subunit vaccine*** | | | | | | | | |
| ZF2001 | Phase 1/2 | Yang, Lancet Infect Dis 2021 | Low | Low | Low | Low | Low | Low |
| SCB-2019 | Phase 1 | Richmond, Lancet 2021 | Low | Low | Low | Low | Low | Low |
| NVX-CoV2373 | Phase 1 | Keech, NEJM 2020 | Low | Low | Low | Low | Low | Low |
| NVX-CoV2373 | Phase 2 | Formica, MedRxiv 2021 | Low | Low | Low | Low | Low | Low |
| EpiVacCorona | Phase 1/2 | Ryzhikov, Russian Journal of Infection and Immunity 2021 | Low | Low | Low | Low | Low | Low |
| MVC-COV1901 | Phase 1 | Hsieh, MedRxiv 2021 | Some concerns | Low | Low | Low | Low | Some concerns |
| ***DNA Vaccine*** | | | | | | | | |
| INO-4800 | Phase 1 | Tebas, EClinicalMedicine 2020 | Some concerns | Low | Low | Low | Low | Some concerns |
| INO-4800 | Phase 2 | Mammen. MedRxiv 2021 | Low | Low | Low | Low | Low | Low |
| ***Virus-like particle vaccine*** | | | | | | | | |
| CoVLP | Phase 1 | Ward, MedRxiv 2020 | Low | Low | Low | Low | Low | Low |

## Table S6. Methodological characteristics of included studies of post-marketing studies: methodological index for non-randomized studies (MINORS) score

| **Vaccine** | **Study ID** | **Clearly stated aim** | **Inclusion of consecutive patients** | **Prospective data collection** | **Endpoints appropriate to study aim** | **Unbiased assessment of study endpoint** | **Follow-up period appropriate to study aim** | **<5% lost to follow-up** | **Prospective calculation of study size** | **Adequate control group** | **Contemporary groups** | **Baseline equivalence of groups** | **Adequate statistical analyses** | **Overall score** |
| --- | --- | --- | --- | --- | --- | --- | --- | --- | --- | --- | --- | --- | --- | --- |
| ***Inactivated vaccine*** | | | | | | | | | | | | | | |
| COVID-19 vaccine (Aikewei) | Wang, Human Vaccines & Immunotherapeutics 2021 | 2 | 2 | 2 | 2 | 2 | 2 | 0 | 0 | - | - | - | - | 12/16 |
| CoronaVac | Zhang, Expert Review of Vaccines2021 | 2 | 2 | 2 | 2 | 1 | 0 | 0 | 0 | - | - | - | - | 9/16 |
| ***RNA vaccine*** | | | | | | | | | | | | | | |
| BNT162b2 | Antonella, EClinicalMedicine 2021 | 2 | 1 | 2 | 2 | 2 | 2 | 0 | 0 | - | - | - | - | 11/16 |
| BNT162b2 &  mRNA-1273 | Chapin-Bardales, Jama 2021 | 2 | 2 | 2 | 2 | 2 | 2 | 2 | 0 | - | - | - | - | 14/16 |
| ***Non-replicating vector vaccine*** | | | | | | | | | | | | | | |
| Covishield vaccine | Konu, MedRxiv 2021 | 2 | 2 | 2 | 2 | 0 | 2 | 0 | 2 | - | - | - | - | 12/16 |
| Gam-COVIDVac | Montalti, MedRxiv 2021 | 2 | 2 | 2 | 2 | 2 | 2 | 2 | 0 | - | - | - | - | 14/16 |
| ChAdOx1-nCoV | Jeon, J Korean Med Sci 2021 | 2 | 2 | 0 | 2 | 2 | 2 | 0 | 0 | - | - | - | - | 10/16 |
| Covishield vaccine, ChAdOx1 nCoV- 19 Corona Virus Vaccine | Kataria, MedRxiv 2021 | 2 | 2 | 2 | 2 | 2 | 2 | 0 | 0 | - | - | - | - | 12/16 |
| ***Multi-manufactures*** | | | | | | | | | | | | | | |
| ChAdOx1-nCoV, BNT162b2 | Menni, Lancet Infect Dis 2021 | 2 | 2 | 2 | 2 | 2 | 2 | 0 | 0 | - | - | - | - | 12/16 |
| ChAdOx1-nCoV, BNT162b2 | Song, J Korean Med Sci 2021 | 2 | 1 | 1 | 2 | 1 | 2 | 0 | 0 | - | - | - | - | 9/16 |
| ChAdOx1-nCoV, BNT162b2 | Kim, J Korean Med Sci 2021 | 2 | 2 | 2 | 2 | 1 | 2 | 0 | 0 | - | - | - | - | 11/16 |
| ChAdOx1-nCoV, BNT162b2 | Bae, J Korean Med Sci 2021 | 2 | 2 | 2 | 2 | 2 | 0 | 0 | 0 | - | - | - | - | 10/16 |

**Methodological items for non-randomized studies:**

**Item 1. A clearly stated aim:** the question addressed should be precise and relevant in the light of available literature

**Item 2. Inclusion of consecutive patients**: all patients potentially fit for inclusion (satisfying the criteria for inclusion) have been included in the study during the study period (no exclusion or details about the reasons for exclusion)

**Item 3. Prospective collection of data**: data were collected according to a protocol established before the beginning of the study

**Item 4. Endpoints appropriate to the aim of the study**: unambiguous explanation of the criteria used to evaluate the main outcome which should be in accordance with the question addressed by the study. Also, the endpoints should be assessed on an intention-to-treat basis.

**Item 5. Unbiased assessment of the study endpoint**: blind evaluation of objective endpoints and double-blind evaluation of subjective endpoints. Otherwise the reasons for not blinding should be stated

**Item 6. Follow-up period appropriate to the aim of the study:** the follow-up should be sufficiently long to allow the assessment of the main endpoint and possible adverse events

**Item 7. Loss to follow up less than 5%:** all patients should be included in the follow up. Otherwise, the proportion lost to follow up should not exceed the proportion experiencing the major endpoint

**Item 8. Prospective calculation of the study size:** information of the size of detectable difference of interest with a calculation of 95% confidence interval, according to the expected incidence of the outcome event, and information about the level for statistical significance and estimates of power when comparing the outcomes

Additional criteria in the case of comparative study:

**Item 9. An adequate control group:** having a gold standard diagnostic test or therapeutic intervention recognized as the optimal intervention according to the available published data

**Item 10. Contemporary groups:** control and studied group should be managed during the same time period (no historical comparison)

**Item 11. Baseline equivalence of groups:** the groups should be similar regarding the criteria other than the studied endpoints. Absence of confounding factors that could bias the interpretation of the results

**Item 12. Adequate statistical analyses:** whether the statistics were in accordance with the type of study with calculation of confidence intervals or relative risk

The items are scored 0 (not reported), 1 (reported but inadequate) or 2 (reported and adequate). The global ideal score being 16 for non-comparative studies and 24 for comparative studies.

## Table S7. Raw data of common AEFIs in the total safety set for candidate vaccines in clinical trials among general population (n/N, %)

| ***Vaccine*** | ***Age range (yrs old)*** | **Dose** | **Severity grade** | **Local reactions** | |  | **Systemic reactions** | |  | **Note** |
| --- | --- | --- | --- | --- | --- | --- | --- | --- | --- | --- |
|  |  |  |  | **Vaccine** | **Placebo** |  | **Vaccine** | **Placebo** |  |  |
| Inactivated vaccines | | | | | | | | | | |
| BBIBP-CorV | 18-59 (Phase 2) | Any dose | Total | 10/84 (11.9) | 1/28 (3.6) |  | 3/84 (3.6) | 3/28 (10.7) |  | - The occurrences of most common reaction were used as a proxy (local reaction/injection pain, systemic reaction/fever). - Incidence of overall adverse events within 30 days after 2 doses was 15/84 in vaccine group and 6/28 in control group. |
|  |  |  | Grade 3 or higher | 0/84 | 0/28 |  | 0/84 | 1/28 (3.6) |  |  |
|  | >=18 (Phase /3) | Any dose | Total | 2786/13471 (20.7) | 3906/13453 (29.0) |  | 3810/13471 (28.3) | 3743/13453 (27.8) |  | - The most common reactions were local reaction/injection pain, systemic reaction/headache. - SAE in appendix Table S8. |
|  |  |  | Grade 3 or higher | 3/13471 (<0.1) | 7/13453 (<0.1) |  | 74/13471 (0.6) | 79/13453 (0.6) |  |  |
| WBIP | 18-59 (Phase 2) | Dose 1 | Total | 7/84 (8.3) | 5/28 (17.9) |  | 3/84 (3.6) | 2/28 (7.1) |  | - Incidence of overall adverse events within 28 days after 2 doses was 16/84 in vaccine group and 5/28 in control group. - No vaccine-related grade 3 reactions were noted. - The most common reactions were local/injection pain (12/84) and systemic reaction/fever (2/84). |
|  |  | Dose 2 | Total | 8/84 (9.5) | 3/28 (10.7) |  | 1/84 (1.2) | 0/28 |  |  |
|  |  | Any dose | Total | 13/84 (15.5) | 4/28 (14.3) |  | 4/84 (4.8) | 2/28 (7.1) |  |  |
|  | >=18 (Phase /3) | Any dose | Total | 3450/13464 (24.3) | 3906/13453 (29.0) |  | 3695/13464 (27.4) | 3743/13453 (27.8) |  | - The most common reactions were local reaction/injection pain, systemic reaction/headache. - SAE in appendix Table S8. |
|  |  |  | Grade 3 or higher | 7/13464 (<0.1) | 7/13453 (<0.1) |  | 84/13464 (0.6) | 79/13453 (0.6) |  |  |
| CoronaVac | 3-17  (Phase 1/2) | Any dose | Total | 35/217 (16) | 2/114 (2) |  | 11/217 (5) | 5/114 (4) |  | - Overall rates of adverse reactions within 28 days in vaccine and placebo groups were 63/217 (29%) and 27/114 (24%). - No grade 3 reactions were noted. - The occurrences of most common reaction were used as a proxy (local reaction/injection pain, systemic reaction/fever). |
|  | 18-59 (Phase 1/2/3) | Dose 1 | Total | 2881/6585 (43.8) | 1431/6436 (22.2) |  | 2064/6585 (31.3) | 2052/6436 (31.9) |  | - Grade 3 adverse events were noted on 98/6202 in phase 3 trial in Brazil. - See appendix Table S8. |
|  |  | Dose 2 | Total | 2606/5811 (44.8) | 1096/5637 (19.4) |  | 1401/5811 (24.1) | 1334/5637 (23.7) |  |  |
|  |  | Any dose | Total | 3972/6560 (60.5) | 3751/6350 (59.1) |  | 3130/6560 (47.7) | 2993/6350 (47.1) |  |  |
|  | >=60 (Phase 1/2/3) | Dose 1 | Total | 12/149 (8.1) | 4/86 (4.7) |  | 17/149 (11.4) | 10/86 (11.6) |  | - No grade 3 or higher reactions were noted. - No vaccine-related serious adverse events were noted within 28 days of vaccination. - The occurrences of most common reaction were used as a proxy (local reaction/injection pain, systemic reaction/headache). |
|  |  | Dose 2 | Total | 14/149 (9.4) | 2/80 (2.5) |  | 5/149 (3.4) | 7/80 (8.8) |  |  |
|  |  | Any dose | Total | 23/150 (15.3) | 5/81 (6.2) |  | 20/150 (13.3) | 15/81 (18.5) |  |  |
| CAMS vaccine | 18-59 (Phase 1/2) | Any dose | Total | 29/174 (16.7) | 6/99 (6.1) |  | 21/174 (12.1) | 11/99 (11.1) |  | - No grade 3 or higher reactions and SAEs were noted. - Incidence of overall adverse events within 28 days was 45/174 in vaccine group and 16/99 in control group. - The most common reactions were local/injection pain (29/174) and systemic/fatigue (10/174). |
| BBV152 | 12-65  (Phase 2) | Dose 1 | Total | 6/190 (3.2) | — |  | 8/190 (4.2) | — |  | - No grade 3 reactions and SAEs were noted. - The occurrences of most common reaction were used as a proxy (local reaction/injection pain, systemic reaction/fever). |
|  |  | Dose 2 | Total | 5/190 (2.6) | — |  | 4/190 (2.1) | — |  |  |
| KCONVAC | 18-59  (Phase 2) | Any dose | Total | 16/100 (16) | 7/50 (14) |  | 6/100 (6) | 2/50 (4) |  | - No grade 3 or higher reactions were noted. - The most common reactions were local/injection pain (15/100) and systemic/headache (2/100). |
| RNA vaccines | | | | | | | | | | |
| BNT162b2 | 12-15  (Phase 3) | Dose 1 | Total | 973/1131 (86) | 260/1129 (23) |  | 679/1131 (60) | 203/1129 (18) |  | - The occurrences of most common reaction were used as a proxy. |
|  |  | Dose 2 | Total | 893/1130 (79) | 440/1129 (39) |  | 746/1130 (66) | 271/1129 (24) |  |  |
|  | 16 and older  (Phase 3) | Dose 1 | Total | 3216/4093 (78.6) | 525/4090 (12.8) |  | 2421/4093 (59.1) | 1922/4090 (47.0) |  | - See appendix Table S8. |
|  |  | Dose 2 | Total | 2748/3758 (69.9) | 396/3749 (10.6) |  | 2627/3758 (69.9) | 1267/3749 (33.8) |  |  |
|  |  | Any dose | Total | 3481/4108 (84.7) | 748/4106 (18.2) |  | 3181/4108 (77.4) | 2255/4106 (54.9) |  |  |
| mRNA-1273 | 18 and older  (Phase 3) | Dose 1 | Total | 12765/15163 (84.2) | 2998/15150 (19.8) |  | 8321/15166 (54.9) | 6398/15154 (42.2) |  | - See appendix Table S8. |
|  |  |  | Grade 3 or higher | 529/15163 (3.5) | 78/15150 (0.5) |  | 452/15166 (3.0) | 315/15154 (2.1) |  |  |
|  |  | Dose 2 | Total | 12381/13944 (88.8) | 2607/13866 (18.8) |  | 11064/13947 (79.3) | 5069/13869 (36.5) |  |  |
|  |  |  | Grade 3 or higher | 978/13944 (7.0) | 70/13866 (5.0) |  | 2200/13947 (15.8) | 276/13869 (2.0) |  |  |
|  |  | Any dose | Total | 13962/15176 (92.0) | 4381/15161 (28.9) |  | 12553/15176 (82.7) | 8032/15162 (53.0) |  |  |
|  |  |  | Grade 3 or higher | 1386/15176 (9.1) | 143/15161 (0.9) |  | 2501/15176 (16.5) | 560/15162 (3.7) |  |  |
| mRNA-1273.351 | 18 and older  (Phase 2) | Dose 3 | Total | 13/20 (65) | 18/20 (90) |  | 11/20 (55) | 16/20 (80) |  | Placebo was mRNA-1273. |
|  |  |  | Grade 3 or higher | 1/20 (5) | 1/20 (5) |  | 1/20 (5) | 3/20 (15) |  |  |
| CVnCoV | 19-59  (Phase 1) | Dose 1 | Total | 26/28 (92.9) | 4/32 (12.5) |  | 27/28 (96.4) | 15/32 (46.9) |  | - The occurrences of most common reaction were used as a proxy (local/injection pain, systemic/fatigue). - 1 withdrawal due to AE. |
|  |  |  | Grade 3 or higher | 0/28 | 0/32 |  | 4/28 (14.3) | 0/32 |  |  |
|  |  | Dose 2 | Total | 22/26 (84.6) | 1/30 (3.3) |  | 25/26 (96.2) | 7/30 (23.3) |  |  |
|  |  |  | Grade 3 or higher | 0/26 | 0/30 |  | 4/26 (15.4) | 0/30 |  |  |
| Non-replicating vector vaccines | | | | | | | | | | |
| ChAdOx1-nCoV | 18 and older  (Phase 3) | Dose 1 | Total | 1839/2580 (71.3) | 1117/2425 (46.1) |  | 1817/2580 (70.4) | 1320/2425 (54.4) |  | - See appendix Table S8. |
|  |  |  | Grade 3 or higher | 210/2580 (8.1) | 112/2425 (4.6) |  | 192/2580 (7.4) | 41/2425 (1.7) |  |  |
|  |  | Dose 2 | Total | 778/1662 (46.8) | 456/1526 (29.9) |  | 741/1662 (44.6) | 545/1526 (35.7) |  |  |
|  |  |  | Grade 3 or higher | 70/1662 (4.2) | 38/1526 (2.5) |  | 37/1662 (2.2) | 27/1526 (1.8) |  |  |
|  |  | Any dose | Total | 1979/2648 (74.7) | 1258/2497 (50.4) |  | 1932/2648 (73.0) | 1488/2497 (59.6) |  |  |
|  |  |  | Grade 3 or higher | 252/2648 (9.5) | 138/2497 (5.5) |  | 221/2648 (8.3) | 63/2497 (2.5) |  |  |
| Ad5 nCoV | 18-83  (Phase 1/2) | Dose 1 | Total | 89/165 (53.9) | 11/126 (8.7) |  | 61/165 (37.0) | 21/126 (16.7) |  | - The occurrences of most common reaction were used as a proxy (local/injection pain, systemic/fatigue). - Incidence of overall adverse events within 28 days of vaccination was 129/165 in vaccine group and 61/126 in control group. |
|  |  |  | Grade 3 or higher | 0/165 | 0/126 |  | 3/165 (1.8) | 0/126 |  |  |
| Gam-COVID-Vac | 18-60 (Phase 1/2) | Any dose | Total | 8/20 (40.0) | — |  | 20/20 (100.0) | — |  | - The occurrences of most common reaction were used as a proxy (local/injection pain, systemic/fever) in phase 1/2. - See appendix Table S8. |
| Ad26.COV2.S | 18-59  (Phase 3) | Dose 1 | Total | 1218/2036 (59.8) | 413/2049 (20.2) |  | 1252/2036 (61.5) | 745/2049 (36.4) |  | - See appendix Table S8. |
|  |  |  | Grade 3 or higher | 18/2036 (0.9) | 4/2049 (0.2) |  | 47/2036 (2.3) | 12/2049 (0.6) |  |  |
|  | 60 and older | Dose 1 | Total | 467/1320 (35.4) | 244/1331 (18.3) |  | 598/1320 (45.3) | 440/1331 (33.1) |  |  |
|  |  |  | Grade 3 or higher | 5/1320 (0.4) | 2/1331 (0.2) |  | 14/1320 (1.1) | 9/1331 (0.7) |  |  |
| Protein subunit vaccines | | | | | | | | | | |
| NVX-CoV2373 | 18-84  (Phase 1/2) | Dose 1 | Total | 131/253 (51.2) | 39/252 (15.5) |  | 112/255 (43.9) | 91/251 (36.3) |  | - One recipient (<1%) had a related severe unsolicited adverse event (acute colitis) that also met the criteria for a serious adverse event. - One recipient (urinary incontinence) discontinued the trial due to an unsolicited adverse event. |
|  |  |  | Grade 3 or higher | 10/253 (0.4) | 0/252 |  | 10/255 (3.9) | 4/251 (1.6) |  |  |
|  |  | Dose 2 | Total | 175/250 (70.0) | 22/242 (9.1) |  | 132/250 (52.8) | 66/241 (27.4) |  |  |
|  |  |  | Grade 3 or higher | 13/250 (5.2) | 0/242 |  | 14/250 (5.6) | 3/241 (1.2) |  |  |
| SCB-2019 | 18-54  (Phase 1) | Dose 1 | Total | 7/16 (43.8) | 1/30 (3.3) |  | 3/16 (18.8) | 3/30 (10.0) |  | - No grade 3 or higher adverse reactions were noted. - No vaccine-related serious adverse events or withdrawals were noted. |
|  |  | Dose 2 | Total | 7/16 (43.8) | 0/30 |  | 5/16 (31.3) | 6/30 (20.0) |  |  |
| ZF2001 | 20-59  (Phase 1/2) | Any dose | Total | 15/170 (8.8) | 5/160 (3.1) |  | 10/170 (5.9) | 8/160 (5.0) |  | - The occurrences of most common reaction were used as a proxy (local/injection pain, systemic/fever). - Incidence of overall adverse events within 28 days of vaccination was 62/170 in vaccine group and 37/160 in control group. |
|  |  |  | Grade 3 or higher | 1/170 (0.6) | 0/160 |  | 0/170 | 0/160 |  |  |
| EPIVACCORONA | 18-60  (Phase 1-2) | Dose 1 | Total | 6/57 (10.5) | 0/43 |  | 1/57 (1.8) | 0/43 |  | - All vaccine-related adverse reactions were mild and local reactogenicity. |
|  |  | Dose 2 | Total | 4/57 (7.0) | 0/43 |  | 0/57 | 0/43 |  |  |
| Virus-like particle vaccines | | | | | | | | | | |
| CoVLP | 18-55 (Phase 1) | Dose 1 | Total | 20/20 (100.0) | — |  | 10/20 (50.0) | — |  | - 5/20 subject experienced Grade 3 AEs from 0 to 7 days after each vaccination and 1 subject did not receive the second vaccination following Grade 3 AE. - 8/20 subjects with at least one unsolicited AEs up to 21 days after last vaccination. - The most common local and systemic reaction were injection pain, fatigue and headache. |
|  |  |  | Grade 3 or higher | 0/20 | — |  | 1/20 (5.0) | — |  |  |
|  |  | Dose 2 | Total | 19/19 (100.0) | — |  | 15/19 (78.9) | — |  |  |
|  |  |  | Grade 3 or higher | 1/19 (5.3) | — |  | 1/19 (5.3) | — |  |  |
| DNA vaccines | | | | | | | | | | |
| INO-4800 | 18-80 (Phase 1-2) | Any dose | Total | 66/167 (39.5) | 11/50 (22) |  | 49/167 (29.3) | 19/50 (38) |  | - Majority AEs were Grade 1 and 2 in severity. - No Grade 4 AEs, no AESIs and no related SAEs were noted. |

## Table S8. Serious adverse events of COVID-19 vaccines by system organ class in phase 3 clinical trials (n/N, %)

| **SAE list (MedDRA 23.1)** | | | **Infections and infestations** | **General disorders and administration site condition** | **Nervous system disorders** | **Musculoskeletal and connective tissue disorders** | **Respiratory, thoracic and mediastinal disorders** | **Cardiac disorders** | **Injury, poisoning and procedural complications** | **Vascular Disorders** | **Reproductive system and breast disorders** | **Gastrointestinal disorders** | **Pregnancy, the puerperium and perinatal conditions** | **Psychiatric disorders** |
| --- | --- | --- | --- | --- | --- | --- | --- | --- | --- | --- | --- | --- | --- | --- |
| **ChAdOx1-nCoV** | **Vaccine** | **(N=12282)** | 23 (0.2) | 4  (<0.1) | 10  (0.1) | 5  (<0.1) | 1  (<0.1) | 7  (<0.1) | 15  (0.1) | 0 | 8  (0.1) | 15  (0.1) | 4 (<0.1) | 3 (<0.1) |
|  | **Placebo** | **(N=11962)** | 41  (0.3) | 2  (<0.1) | 8  (0.1) | 3  (<0.1) | 1  (<0.1) | 10  (0.1) | 17  (0.1) | 2  (<0.1) | 2  (<0.1) | 13  (0.1) | 2 (<0.1) | 1 (<0.1) |
| **Gam-COVID-Vac** | **Vaccine** | **(N=16427)** | 8  (<0.1) | 1  (<0.1) | 2  (<0.1) | 1  (<0.1) | 0 | 4  (<0.1) | 6  (<0.1) | 10  (0.1) | 3  (<0.1) | 4  (<0.1) | 0 | 0 |
|  | **Placebo** | **(N=5435)** | 14  (0.3) | 0 | 1  (<0.1) | 0 | 1  (<0.1) | 1  (<0.1) | 0 | 3  (0.1) | 2  (<0.1) | 0 | 1 (<0.1) | 0 |
| **BNT162b2** | **Vaccine** | **(N=21621)** | 27  (0.1) | 4/18801 (<0.1) | 18  (0.1) | 3/18801 (<0.1) | 5/18801 (<0.1) | 18  (0.1) | NA | NA | NA | NA | NA | NA |
|  | **Placebo** | **(N=21631)** | 17  (0.1) | 3/18785 (<0.1) | 16  (0.1) | 1/18785 (<0.1) | 4/18785 (<0.1) | 18  (0.1) | NA | NA | NA | NA | NA | NA |
| **CoronaVac** | **Vaccine** | **(N=6202)** | 13  (0.2) | 3  (0.1) | 1  (<0.1) | 3  (0.1) | 3  (0.1) | 0 | 4  (0.1) | 2  (<0.1) | 0 | 1  (<0.1) | 1 (<0.1) | 3  (0.1) |
|  | **Placebo** | **(N=6194)** | 13  (0.2) | 0 | 1  (<0.1) | 0 | 0 | 1  (<0.1) | 5  (0.1) | 0 | 1  (<0.1) | 1  (<0.1) | 3  (0.1) | 2 (<0.1) |
| **Sinopharm** | **BBIBP-CorV** | **(N=13471)** | 20  (0.2) | 2  (<0.1) | 5 (<0.1) | 5  (<0.1) | 1  (<0.1) | 9  (0.1) | 11  (0.1) | 5 (<0.1) | 0 | 7  (0.1) | 2 (<0.1) | 2 (<0.1) |
|  | **WBIP** | **(N=13464)** | 21  (0.2) | 3  (<0.1) | 3 (<0.1) | 8  (0.1) | 4  (<0.1) | 0 | 8  (0.1) | 4 (<0.1) | 0 | 8  (0.1) | 0 | 2 (<0.1) |
|  | **Placebo** | **(N=13453)** | 39  (0.3) | 2  (<0.1) | 8  (0.1) | 4  (<0.1) | 5  (<0.1) | 4 (<0.1) | 5  (<0.1) | 1 (<0.1) | 2 (<0.1) | 8  (0.1) | 0 | 2 (<0.1) |

## Table S9. Serious safety outcomes of vaccines in phase 3 clinical trials

| **Vaccine** | **SAE** | |  | **Withdrawal** | |  | **Medically attended** | |  | **Death** | |
| --- | --- | --- | --- | --- | --- | --- | --- | --- | --- | --- | --- |
|  | **Total** | **Related** |  | **Total** | **Related** |  | **Total** | **Related** |  | **Total** | **Related** |
| Platform: RNA | | | | | | | | | | | |
| BNT162b2- Adolescents (12-15 yr) (N=1131) | 4 (0.4) | 0 |  | 2 (0.2) | 1 (0.1) |  | — | — |  | 0 | 0 |
| Placebo (N=1129) | 1 (0.1) | 0 |  | 0 | 0 |  | — | — |  | 0 | 0 |
| BNT162b2-Adults (>=16 yr) (N=21621) | 126 (0.6) | 4 (<0.1) |  | 37 (0.2) | 16 (0.1) |  | — | — |  | 2 (<0.1) | 0 |
| Placebo (N=21631) | 111 (0.5) | 0 |  | 30 (0.1) | 9 (<0.1) |  | — | — |  | 4 (<0.1) | 0 |
| mRNA-1273 (N=15185) | 93 (0.6) | 6 (<0.1) |  | 50 (0.3) | 18 (0.1) |  | 1372 (9.0) | 140 (0.9) |  | 2 (<0.1) | 0 |
| Placebo (N=15166) | 89 (0.6) | 4 (<0.1) |  | 80 (0.5) | 15 (0.1) |  | 1465 (9.7) | 83 (0.5) |  | 3 (<0.1) | 0 |
| Platform: Non-replicating vector | | | | | | | | | | | |
| ChAdOx1-nCoV (N=12021) | 79 (0.7) | 3 (<0.1) |  | — | — |  | — | — |  | 2 (<0.1) | 0 |
| Placebo (N=11724) | 89 (0.8) | 2 (<0.1) |  | — | — |  | — | — |  | 4 (<0.1) | 0 |
| Gam-COVID-Vac (N=16427) | 45 (0.3) | 0 |  | — | — |  | — | — |  | 4 (<0.1) | 0 |
| Placebo (N=5435) | 23 (0.4) | 0 |  | — | — |  | — | — |  | 1 (<0.1) | 0 |
| Ad26.COV2.S (N=21895) | 83 (0.4) | 7 (<0.1) |  | 0 | 0 |  | 304 (1.4) | 22 (0.1) |  | 3 (<0.1) | 0 |
| Placebo (N=21888) | 96 (0.4) | 2 (<0.1) |  | 0 | 0 |  | 408 (1.8) | 22 (0.1) |  | 16 (0.1) | 0 |
| Platform: Inactivated | | | | | | | | | | | |
| CoronaVac (N=6202) | 33 (0.5) | 0 |  | 0 | 0 |  | — | — |  | 1 (<0.1) | 0 |
| Placebo (N=6194) | 31 (0.5) | 0 |  | 0 | 0 |  | — | — |  | 1 (<0.1) | 0 |
| BBIBP-CorV (N=13471) | 59 (0.4) | 2 (<0.1) |  | — | — |  | — | — |  | 0 | 0 |
| WBIP (N=13464) | 64 (0.5) | 0 |  | — | — |  | — | — |  | 0 | 0 |
| Placebo (N=13453) | 78 (0.6) | 0 |  | — | — |  | — | — |  | 0 | 0 |

SAE: serious adverse event.

## Table S10. Summary of unbalanced AESIs between intervention and control groups in phase 3 clinical trials of mRNA vaccines

|  | **BNT162b2 (>=16 yr)** | | | **BNT162b2 (12-15 yr)** | | | **mRNA-1273** | | |
| --- | --- | --- | --- | --- | --- | --- | --- | --- | --- |
| **Group** | Vaccine  (N=21621) | Placebo  (21631) | RR  (95% CI) | Vaccine  (N=1131) | Placebo  (N=1129) | RR  (95% CI) | Vaccine  (N=15184) | Placebo  (N=15165) | RR  (95% CI) |
| Hypersensitivity | 137 (0.6) | 111 (0.5) | **1.2**  **(1.0-1.6)** | 0 | 0 | **—** | 233 (1.5) | 166 (1.1) | **1.4**  **(1.2-1.7)** |
| Lymphadenopathy | 64 (0.3) | 6 (0.6) | **10.7**  **(4.6-24.6)** | 9 (0.8) | 2 (0.2) | **4.5**  **(1.0-20.7)** | 173 (1.1) | 95 (0.6) | **1.8**  **(1.4-2.3)** |
| Bell’s palsy | 4 (<0.01) | 0 | 9.0  (0.5-167.2) | 0 | 0 | **—** | 3 (<0.01) | 1  (<0.01) | 3  (0.3-28.8) |

## Table S11. Age group comparison of most common adverse reactions and fever within 7 days post-vaccination between younger adults and elderly (n/N, %)

| **BNT162b2** | **Vaccine** | **Placebo** | **RR_1_**  **(95%CI)** |  | **Vaccine** | **Placebo** | **RR_2_**  **(95%CI)** |
| --- | --- | --- | --- | --- | --- | --- | --- |
| Age range of participants | 18-55 years and older | | |  | 55 years and older | | |
| Local - Injection pain |  | |  |  |  | |  |
| Dose #1 | 1904/2238  (85.1) | 322/2248  (14.3) | 5.9  (5.4-6.6) |  | 1282/1802  (71.1) | 166/1792  (9.3) | 7.7  (6.6-8.9) |
| Grade 3 or 4 | 24/2238  (1.1) | 2/2248  (0.1) | 12.1  (2.9-50.9) |  | 4/1802  (0.2) | 0/1792  (0.0) | 9.0  (0.5-166.1) |
| Dose #2 | 1632/2045  (79.8) | 245/2053  (11.0) | 6.7  (5.9-7.5) |  | 1098/1660  (66.1) | 127/1646  (7.7) | 8.6  (7.2-10.2) |
| Grade 3 or 4 | 25/2045  (1.2) | 0/2053  (0.0) | 51.2  (3.1-840.4) |  | 8/1660  (0.5) | 0/1646  (0.0) | 16.9  (1.0-291.8) |
| Systemic – Fatigue |  |  |  |  |  |  |  |
| Dose #1 | 1085/2238  (48.5) | 767/2248  (34.1) | 1.4  (1.3-1.5) |  | 615/1802  (34.1) | 405/1792  (20.5) | 5.8  (4.8-7.1) |
| Grade 3 or 4 | 33/2038  (1.5) | 11/2248  (0.5) | 3.0  (1.5-5.9) |  | 2/1802  (0.1) | 3/1792  (0.2) | 0.7  (0.1-4.0) |
| Dose #2 | 1247/2045  (61.0) | 479/2053  (23.3) | 2.6  (2.4-2.8) |  | 839/1660  (50.5) | 277/1646  (16.8) | 3.0  (2.7-3.4) |
| Grade 3 or 4 | 97/2045  (4.7) | 14/2053  (0.7) | 7.0  (4.0-12.1) |  | 46/1660  (2.8) | 2/1646  (0.1) | 22.8  (5.5-93.8) |
| Systemic – Headache |  |  |  |  |  |  |  |
| Dose #1 | 959/2238  (42.9) | 775/2248  (34.5) | 1.2  (1.2-1.3) |  | 454/1802  (25.2) | 325/1792  (16.5) | 2.3  (2.1-2.6) |
| Grade 3 or 4 | 23/2238  (1.0) | 19/2248  (0.8) | 1.2  (0.7-2.2) |  | 2/1802  (0.1) | 3/1792  (0.2) | 0.7  (0.1-4.0) |
| Dose #2 | 1085/2045  (53.1) | 506/2053  (24.6) | 2.2  (2.0-2.3) |  | 647/1660  (39.0) | 229/1646  (13.9) | 2.8  (2.4-3.2) |
| Grade 3 or 4 | 67/2045  (3.3) | 15/2053  (0.7) | 4.5  (2.6-7.8) |  | 9/1660  (0.5) | 4/1646  (0.2) | 2.2  (0.7-7.2) |
| Systemic – Fever |  |  |  |  |  |  |  |
| Dose #1 | 85/2238  (3.8) | 20/2248  (0.9) | 4.3  (2.6-6.9) |  | 26/1802  (1.4) | 7/1792  (0.4) | 6.2  (2.7-14.1) |
| Grade 3 or 4 | 6/2238  (0.3) | 5/2248  (0.2) | 1.2  (0.4-3.9) |  | 2/1802  (0.1) | 2/1792  (0.1) | 1.0  (0.1-7.1) |
| Dose #2 | 331/2045  (16.2) | 10/2053  (0.5) | 33.2  (17.8-62.1) |  | 181/1660  (10.9) | 4/1646  (0.2) | 44.9  (16.7-120.6) |
| Grade 3 or 4 | 27/2045  (1.3) | 2/2053  (0.1) | 13.6  (3.2-56.9) |  | 5/1660  (0.3) | 1/1646  (0.1) | 5.0  (0.6-42.4) |
| **mRNA-1273** | **Vaccine** | **Placebo** | **RR_1_**  **(95%CI)** |  | **Vaccine** | **Placebo** | **RR_2_**  **(95%CI)** |
| Age range of participants | 18-64 years old | | |  | 65 years and older | | |
| Local - Injection pain |  | |  |  |  | |  |
| Dose #1 | 9908/11401  (86.9) | 2179/11404  (19.1) | 4.5  (4.4-4.7) |  | 2782/3762  (74.0) | 481/3746  (12.8) | 5.8  (5.3-6.3) |
| Grade 3 or 4 | 367/11401  (3.2) | 23/11404  (0.2) | 16.0  (10.5-24.3) |  | 50/3762  (1.3) | 32/3746  (0.9) | 1.6  (1.0-2.4) |
| Dose #2 | 9335/10357  (90.1) | 1942/10317  (18.8) | 4.8  (4.6-5.0) |  | 2990/3587  (83.4) | 421/3549  (11.9) | 7.0  (6.4-7.7) |
| Grade 3 or 4 | 479/10357  (4.6) | 21/10317  (0.2) | 22.7  (14.7-35.1) |  | 96/3587  (2.7) | 17/3549  (0.5) | 5.6  (3.3-9.3) |
| Systemic – Fatigue |  |  |  |  |  |  |  |
| Dose #1 | 4384/11401  (38.5) | 3282/11404  (28.8) | 1.3  (1.3-1.4) |  | 1251/3761  (33.3) | 851/3745  (22.7) | 1.5  (1.4-1.6) |
| Grade 3 or 4 | 121/11401  (1.1) | 83/11404  (0.7) | 1.5  (1.1-1.9) |  | 30/3761  (0.8) | 23/3745  (0.6) | 1.3  (0.8-2.2) |
| Dose #2 | 7002/10357  (67.6) | 2530/10315  (24.5) | 2.8  (2.7-2.9) |  | 2094/3587  (58.4) | 695/3549  (19.6) | 3.0  (2.8-3.2) |
| Grade 3 or 4 | 1099/10357  (10.6) | 81/10315  (0.8) | 13.5  (10.8-16.9) |  | 248/3587  (6.9) | 20/3549  (0.6) | 12.3  (7.8-19.3) |
| Systemic – Headache |  |  |  |  |  |  |  |
| Dose #1 | 4031/11401  (35.4) | 3303/11404  (29.0) | 1.2  (1.2-1.3) |  | 921/3761  (24.5) | 724/3745  (19.3) | 1.3  (1.2-1.4) |
| Grade 3 or 4 | 219/11401  (1.9) | 162/11404  (1.4) | 1.4  (1.1-1.7) |  | 52/3761  (1.4) | 34/3745  (0.9) | 1.5  (1.0-2.3) |
| Dose #2 | 6500/10357  (62.8) | 515/10317  (5.0) | 12.6  (11.5-13.7) |  | 1665/3587  (46.4) | 635/3549  (17.9) | 2.6  (2.4-2.8) |
| Grade 3 or 4 | 2617/10357  (25.3) | 124/10317  (1.2) | 21.0  (17.6-25.1) |  | 107/3587  (3.0) | 32/3549  (0.9) | 3.3  (2.2-4.9) |
| Systemic – Fever |  |  |  |  |  |  |  |
| Dose #1 | 105/11403  (0.9) | 39/11404  (0.3) | 2.7  (1.9-3.9) |  | 10/3760  (0.3) | 7/3748  (0.2) | 1.4  (0.5-3.7) |
| Grade 3 or 4 | 14/11403  (0.1) | 5/11404  (<0.1) | 2.8  (1.0-7.8) |  | 1/3760  (0.0) | 3/3748  (0.1) | 0.3  (0.0-3.2) |
| Dose #2 | 1806/10352  (17.4) | 38/10315  (0.4) | **47.3**  **(34.4-65.2)** |  | 366/3587  (10.2) | 5/3549  (0.1) | **72.4**  **(30.0-174.8)** |
| Grade 3 or 4 | 178/10352  (1.7) | 3/10315  (<0.1) | **59.1**  **(18.9-184.9)** |  | 19/3587  (0.5) | 1/3549  (0.0) | **18.8**  **(2.5-140.3)** |
| **ChAdOx1-nCoV*** | **Vaccine** | **Placebo** | **RR_1_**  **(95%CI)** |  | **Vaccine** | **Placebo** | **RR_2_**  **(95%CI)** |
| Age range of participants | 18-55 years old | | |  | 56 years and older | | |
| Local - Tenderness |  | |  |  |  | |  |
| Dose #1 | 73/99  (73.7) | — | — |  | 67/155  (43.2) | — | — |
| Grade 3 or 4 | 1/99  (1.0) | — | — |  | 0/155  (0.0) | — | — |
| Dose #2 | 60/99  (60.6) | — | — |  | 61/153  (39.9) | — | — |
| Grade 3 or 4 | 0/99  (0.0) | — | — |  | 0/153  (0.0) | — | — |
| Systemic – Fatigue |  |  |  |  |  |  |  |
| Dose #1 | 64/99  (64.6) | — | — |  | 60/155  (38.7) | — | — |
| Grade 3 or 4 | 4/99  (4.0) | — | — |  | 0/155  (0.0) | — | — |
| Dose #2 | 45/100  (45.0) | — | — |  | 45/153  (29.4) | — | — |
| Grade 3 or 4 | 1/100  (1.0) | — | — |  | 0/153  (0.0) | — | — |
| Systemic – Headache |  |  |  |  |  |  |  |
| Dose #1 | 53/99  (53.5) | — | — |  | 50/155  (32.3) | — | — |
| Grade 3 or 4 | 2/99  (2.0) | — | — |  | 0/155  (0.0) | — | — |
| Dose #2 | 28/100  (28.0) | — | — |  | 28/153  (18.3) | — | — |
| Grade 3 or 4 | 1/100  (1.0) | — | — |  | 0/153  (0.0) | — | — |
| Systemic - Fever |  |  |  |  |  |  |  |
| Dose #1 | 12/99  (12.1) | — | — |  | 4/155  (2.6) | — | — |
| Grade 3 or 4 | 0/99  (0.0) | — | — |  | 1/155  (0.6) | — | — |
| Dose #2 | 0/100  (0.0) | — | — |  | 1/153  (0.7) | — | — |
| Grade 3 or 4 | 0/100  (0.0) | — | — |  | 0/153  (0.0) | — | — |
| **Ad26.COV2.S** | **Vaccine** | **Placebo** | **RR_1_**  **(95%CI)** |  | **Vaccine** | **Placebo** | **RR_2_**  **(95%CI)** |
| Age range of participants | 18-59 years old | | |  | 60 years and older | | |
| Local – Injection pain |  | |  |  |  | |  |
| Dose #1 | 1193/2036  (58.6) | 357/2049  (17.4) | **3.4**  **(3.0-3.7)** |  | 439/1320  (33.3) | 207/1331  (15.6) | **2.1**  **(1.8-2.5)** |
| Grade 3 or 4 | 8/2036  (0.4) | 0/2049  (0.0) | **17.1**  **(1.0-296.2)** |  | 3/1320  (0.2) | 2/1331  (0.2) | 1.5  (0.3-9.0) |
| Systemic – Fatigue |  |  |  |  |  |  |  |
| Dose #1 | 891/2036  (43.8) | 451/2049  (22.0) | **2.0**  **(1.8-2.2)** |  | 392/1320  (29.7) | 277/1331  (20.8) | **1.4**  **(1.2-1.6)** |
| Grade 3 or 4 | 25/2036  (1.2) | 4/2049  (0.2) | **6.3**  **(2.2-18.0)** |  | 10/1320  (0.8) | 5/1331  (0.4) | 2.0  (0.7-5.9) |
| Systemic – Headache |  |  |  |  |  |  |  |
| Dose #1 | 905/2036  (44.4) | 508/2049  (24.8) | **1.8**  **(1.6-2.0)** |  | 401/1320  (30.4) | 294/1331  (22.1) | **1.4**  **(1.2-1.6)** |
| Grade 3 or 4 | 18/2036  (0.9) | 5/2049  (0.2) | **3.6**  **(1.3-9.7)** |  | 5/1320  (0.4) | 4/1331  (0.3) | 1.3  (0.3-4.7) |
| Systemic – Fever |  |  |  |  |  |  |  |
| Dose #1 | 261/2036  (12.8) | 14/2049  (0.7) | **18.8**  **(11.0-32.2)** |  | 41/1320  (3.1) | 6/1331  (0.5) | **6.9**  **(2.9-16.2)** |
| Grade 3 or 4 | 7/2036  (0.3) | 0/2049  (0.0) | 15.1  (0.9-264.1) |  | 1/1320  (0.1) | 0/1320  (0.0) | 3.0  (0.1-74.2) |
| **NVX-CoV2373** | **Vaccine** | **Placebo** | **RR_1_**  **(95%CI)** |  | **Vaccine** | **Placebo** | **RR_2_**  **(95%CI)** |
| Age range of participants | 18-59 years old | | |  | 60-84 years old | | |
| Local - Tenderness |  | |  |  |  | |  |
| Dose #1 | 83/139  (59.7) | 21/138  (15.2) | **3.9**  **(2.6-6.0)** |  | 39/114  (34.2) | 12/114  (10.5) | **3.2**  **(1.8-5.9)** |
| Grade 3 or 4 | 1/139  (0.7) | 0/138 | 3.0  (0.1-72.5) |  | 0/114 | 0/114 | — |
| Dose #2 | 101/137  (73.7) | 12/132  (9.0) | **8.1**  **(4.7-14.0)** |  | 62/113  (54.9) | 6/110  (5.5) | **10.1**  **(4.5-22.3)** |
| Grade 3 or 4 | 9/137  (6.6) | 0/132 | **18.3**  **(1.1-311.4)** |  | 0/113 | 0/110 | — |
| Systemic – Fatigue |  |  |  |  |  |  |  |
| Dose #1 | 37/139  (26.5) | 32/138  (23.2) | 1.1  (0.8-1.7) |  | 22/116  (19.0) | 20/113  (17.7) | 1.1  (0.6-1.9) |
| Grade 3 or 4 | 3/139  (2.3) | 1/138  (0.7) | 3.0  (0.3-28.3) |  | 2/116  (1.7) | 0/113 | 4.9  (0.2-100.4) |
| Dose #2 | 63/137  (46.0) | 22/132  (16.7) | 2.8  (1.8-4.2) |  | 26/113  (23.0) | 11/109  (10.1) | **2.3**  **(1.2-4.4)** |
| Grade 3 or 4 | 7/137  (5.1) | 0/132 | 14.5  (0.8-250.6) |  | 0/113 | 1/109  (0.9) | 0.3  (0.0-7.8) |
| Systemic – Headache |  |  |  |  |  |  |  |
| Dose #1 | 40/139  (28.7) | 35/138  (25.4) | 1.1  (0.8-1.7) |  | 19/116  (16.4) | 17/113  (15.0) | 1.1  (0.6-2.0) |
| Grade 3 or 4 | 5/139  (3.7) | 1/138  (0.7) | 5.0  (0.6-41.9) |  | 0/116 | 0/113 | — |
| Dose #2 | 68/137  (49.9) | 19/132  (14.5) | 3.4  (2.2-5.4) |  | 6/113  (5.0) | 12/109  (11.0) | 0.5  (0.2-1.2) |
| Grade 3 or 4 | 4/137  (2.9) | 1/132  (0.7) | 3.9  (0.4-34.0) |  | 1/113  (0.9) | 0/109 | 2.9  (0.1-70.3) |
| Systemic - Fever |  |  |  |  |  |  |  |
| Dose #1 | 2/139  (1.6) | 3/136  (2.2) | 0.7  (0.1-3.8) |  | 4/116  (3.4) | 3/112  (2.7) | 1.3  (0.3-5.6) |
| Grade 3 or 4 | 1/139  (0.8) | 0/136 | 2.9  (0.1-71.4) |  | 2/116  (1.7) | 0/112 | 4.8  (0.2-99.5) |
| Dose #2 | 9/137  (6.5) | 1/130  (0.7) | **8.5**  **(1.1-66.5)** |  | 2/112  (1.8) | 1/109  (0.9) | 1.9  (0.2-21.2) |
| Grade 3 or 4 | 1/137  (0.7) | 0/130 | 2.8  (0.1-69.3) |  | 0/112 | 0/109 | — |
| **CoronaVac** | **Vaccine** | **Placebo** | **RR_1_**  **(95%CI)** |  | **Vaccine** | **Placebo** | **RR_2_**  **(95%CI)** |
| Age range of participants | 18-59 years old | | |  | 60 years and older | | |
| Local – Injection pain |  | |  |  |  | |  |
| Dose #1 | 2878/6585  (43.7) | 1431/6436  (22.2) | **2.0**  **(1.9-2.1)** |  | 12/149  (8.1) | 4/86  (4.7) | 1.7  (0.6-5.2) |
| Dose #2 | 2605/5811  (44.8) | 1096/5637  (19.4) | **2.3**  **(2.2-2.4)** |  | 13/149  (8.7) | 2/80  (2.5) | 3.5  (0.8-15.1) |
| Any dose | 3896/6560  (59.4) | 2051/6350  (32.3) | **1.8**  **(1.8-1.9)** |  | 22/150  (14.7) | 5/81  (6.2) | 2.4  (0.9-6.0) |
| Systemic – Headache |  |  |  |  |  |  |  |
| Dose #1 | 2049/6585  (31.1) | 2045/6436  (31.8) | 1.0  (0.9-1.0) |  | 5/149  (3.4) | 1/86  (1.2) | 2.9  (0.3-24.3) |
| Dose #2 | 1399/5811  (24.1) | 1329/5637  (23.6) | 1.0  (1.0-1.1) |  | 4/149  (2.7) | 3/80  (3.8) | 0.7  (0.2-3.1) |
| Any dose | 2240/6560  (34.1) | 2193/6350  (34.5) | 1.0  (0.9-1.0) |  | 7/150  (4.7) | 3/81  (3.7) | 1.3  (0.3-4.7) |
| Systemic – Fatigue |  |  |  |  |  |  |  |
| Dose #1 | 919/6585  (14.0) | 834/6436  (13.0) | 1.1  (1.0-1.2) |  | 7/149  (4.7) | 1/86  (1.2) | 4.0  (0.5-32.3) |
| Dose #2 | 521/5811  (9.0) | 550/5634  (9.8) | 0.9  (0.8-1.0) |  | 2/149  (1.3) | 3/80  (3.8) | 0.4  (0.1-2.1) |
| Any dose | 1055/6560  (16.1) | 949/6350  (14.9) | 1.1  (1.0-1.2) |  | 8/150  (5.3) | 3/81  (3.7) | 1.4  (0.4-5.3) |
| Systemic – Fever |  |  |  |  |  |  |  |
| Dose #1 | 11/6585  (0.2) | 10/6436  (0.2) | 1.1  (0.5-2.5) |  | 3/149  (2.0) | 1/86  (1.2) | 1.7  (0.2-16.4) |
| Dose #2 | 5/5811  (0.1) | 4/5637  (0.1) | 1.2  (0.3-4.5) |  | 1/149  (0.7) | 0/80 | 1.6  (0.1-39.2) |
| Any dose | 14/6560  (0.2) | 6/6350  (0.1) | 2.3  (0.9-5.9) |  | 4/150  (2.7) | 1/81  (1.2) | 2.2  (0.2-19.0) |
| **SCB-2019** | **Vaccine** | **Placebo** | **RR_1_**  **(95%CI)** |  | **Vaccine** | **Placebo** | **RR_2_**  **(95%CI)** |
| Age range of participants | 18-54 years old | | |  | 55-75 years old | | |
| Local – Injection pain |  | |  |  |  | |  |
| Dose #1 | 4/8  (50.0) | 1/18  (5.6) | **9.0**  **(1.2-68.3)** |  | 1/8  (12.5) | 0/12 | 4.4  (0.2-96.0) |
| Dose #2 | 5/8  (62.5) | 0/18 | **23.9**  **(1.5-385.4)** |  | 2/8  (25.0) | 0/12 | 7.4  (0.4-134.8) |
| Systemic – Fatigue |  |  |  |  |  |  |  |
| Dose #1 | 0/8 | 0/18 | — |  | 0/8 | 0/12 | — |
| Dose #2 | 1/8  (12.5) | 1/18  (5.6) | 2.2  (0.2-31.6) |  | 1/8  (12.5) | 1/12  (8.3) | 1.5  (0.1-20.7) |
| Systemic – Headache |  |  |  |  |  |  |  |
| Dose #1 | 3/8  (37.5) | 1/18  (5.6) | 6.8  (0.8-55.4) |  | 0/8 | 0/12 | — |
| Dose #2 | 2/8  (25.0) | 1/18  (5.6) | 4.5  (0.5-42.8) |  | 0/8 | 2/12  (16.7) | 0.3  (0.0-5.4) |

*: Adverse reactions in groups receiving either low and standard doses were combined.

## Table S12. Meta-analyses for comparing the rates of most common AEFI of COVID-19 candidate vaccines versus placebo or control vaccine by platform among younger adults (18-65 years old)

| Treatment comparison  (reference: placebo) | Study group (n/N) | | Pooled RR  (95%CI) |
| --- | --- | --- | --- |
|  | Treatment | Control |  |
| Injection site pain, *15 vaccines on 5 platforms* | | | |
| Inactivated vaccines | 9825/33769 | 5821/19952 | 1.2 (0.7-2.0) |
| RNA vaccines | 11265/12623 | 2268/12597 | **5.4 (4.4-6.6)** |
| Non-replicating vector vaccines | 2419/13590 | 885/13408 | **3.0 (2.2-4.0)** |
| Protein subunit vaccines | 136/436 | 16/442 | **7.1 (2.4-20.5)** |
| DNA vaccines | 41/147 | 9/51 | 1.6 (0.8-3.0) |
| Fatigue, *15 vaccines on 5 platforms* | | | |
| Inactivated vaccines | 4043/33769 | 2381/19952 | 1.1 (1.0-1.1) |
| RNA vaccines | 8276/12430 | 3024/12400 | **2.7 (2.5-2.9)** |
| Non-replicating vector vaccines | 2751/13590 | 1704/13408 | **1.7 (1.4-2.0)** |
| Protein subunit vaccines | 92/436 | 39/431 | 1.7 (0.6-5.0) |
| DNA vaccines | 48/147 | 19/51 | 0.9 (0.6-1.3) |
| Headache, *15 vaccines on 5 platforms* | | | |
| Inactivated vaccines | 5736/33769 | 3887/19952 | 1.0 (1.0-1.0) |
| RNA vaccines | 7610/12430 | 1031/12442 | 4.3 (0.9-19.4) |
| Non-replicating vector vaccines | 2750/13590 | 1794/13408 | **1.6 (1.3-1.8)** |
| Protein subunit vaccines | 79/436 | 33/431 | **2.3 (1.6-3.4)** |
| DNA vaccines | 43/147 | 16/50 | 0.9 (0.6-1.5) |
| Fever, *14 vaccines on 4 platforms* | | | |
| Inactivated vaccines | 569/33769 | 284/19952 | 1.0 (0.9-1.2) |
| RNA vaccines | 2153/12425 | 48/12400 | **43.9 (33.1-58.3)** |
| Non-replicating vector vaccines | 546/13590 | 63/13408 | **6.2 (2.4-16.5)** |
| Protein subunit vaccines | 21/435 | 10/429 | 2.2 (0.5-9.7) |

AEFI = Adverse event following immunization; n = number of participants experiencing at least one AEFI; N = total number of participants included in analysis; CI = Confidence Intervals. Per-protocol analysis.

## Table S13. Multivariate meta-regression determining factors accounting for the heterogeneity of safety profile

| **Outcome** | **Study characteristics** | **β coefficient** | **SE** | **P value** |
| --- | --- | --- | --- | --- |
| Injection site pain | | | | |
|  | Platform |  |  |  |
|  | Inactivated | 1.0 | — | — |
|  | RNA | 0.66 | 0.10 | <0.01 |
|  | Non-replicating vector | 0.24 | 0.10 | <0.01 |
|  | Protein subunit | 0.26 | 0.11 | 0.01 |
|  | Virus-like particle | 0.76 | 0.19 | <0.01 |
|  | DNA | 0.04 | 0.19 | 0.82 |
|  | Age group |  |  |  |
|  | Adults (aged under 65 years old) | 1.0 | — | — |
|  | Elderly (aged 65 years and older) | -0.13 | 0.08 | 0.13 |
| Fatigue |  |  |  |  |
|  | Platform |  |  |  |
|  | Inactivated | 1.0 | — | — |
|  | RNA | 0.61 | 0.09 | <0.01 |
|  | Non-replicating vector | 0.29 | 0.09 | <0.01 |
|  | Protein subunit | 0.14 | 0.10 | 0.17 |
|  | Virus-like particle | 0.49 | 0.20 | 0.01 |
|  | DNA | 0.24 | 0.17 | 0.16 |
|  | Age group |  |  |  |
|  | Adults (aged under 65 years old) | 1.0 | — | — |
|  | Elderly (aged 65 years and older) | -0.07 | 0.08 | 0.36 |
| Headache |  |  |  |  |
|  | Platform |  |  |  |
|  | Inactivated | 1.0 | — | — |
|  | RNA | 0.52 | 0.08 | <0.01 |
|  | Non-replicating vector | 0.32 | 0.08 | <0.01 |
|  | Protein subunit | 0.14 | 0.09 | 0.11 |
|  | Virus-like particle | 0.46 | 0.18 | 0.01 |
|  | DNA | 0.17 | 0.15 | 0.25 |
|  | Age group |  |  |  |
|  | Adults (aged under 65 years old) | 1.0 | — | — |
|  | Elderly (aged 65 years and older) | -0.16 | 0.07 | 0.02 |
| Fever |  |  |  |  |
|  | Platform |  |  |  |
|  | Inactivated | 1.0 | — | — |
|  | RNA | 0.23 | 0.12 | 0.05 |
|  | Non-replicating vector | 0.26 | 0.11 | 0.02 |
|  | Protein subunit | 0.05 | 0.12 | 0.67 |
|  | Virus-like particle | 0.32 | 0.24 | 0.17 |
|  | DNA |  |  |  |
|  | Age group |  |  |  |
|  | Adults (aged under 65 years old) | 1.0 | — | — |
|  | Elderly (aged 65 years and older) | -0.15 | 0.09 | 0.10 |

## Table S14. Summary of post-authorization active surveillance studies among general population

| **First Author** | **Country** | **Follow-up period** | **Subjects** | **Vaccine** | **No. of subjects** | **Age** | **Characteristics** | **AEFI** | **Local pain** | **Fatigue** | **Headache** | **Fever** | **Note** |
| --- | --- | --- | --- | --- | --- | --- | --- | --- | --- | --- | --- | --- | --- |
| Seongman Bae | South Korea | 3 days after dose 1 | HCWs | BNT162b2 | 277 | 20-69 | NA | 222 (80.1%) | 194 (70.0%) | 104 (37.5%) | 67 (24.2%) | 19 (6.9%) | No significant difference in the frequency of adverse reactions according to sex, and the number of categories showing a significant trend according to age group was small. |
|  |  |  |  | ChAdOx1  nCoV-19 | 5589 | 20-69 | NA | 5217 (93.3%) | 4536 (81.2%) | 4277 (76.5%) | 3887 (69.5%) | 2865 (51.3%) | Adverse reactions were significantly more frequent in females and in younger age groups. |
| Johanna Chapin-Bardales | United States | Days 0 to 7 after each dose | GP | BNT162b2 | Dose 1: 1659724; Dose 2: 971375 | >=16 | NA | NA | Dose 1: 1055604 (63.6%);  Dose 2: 645917 (66.5%) | Dose 1: 489146 (29.1%);  Dose 2: 464659 (47.8%) | Dose 1: 409359 (24.7%);  Dose 2: 392266 (40.4%) | Dose 1: 116951 (7.0%);  Dose 2:  208976 (21.5%) | NA |
|  |  |  |  | mRNA-1273 | Dose 1: 1984194; Dose 2: 949497 | >=18 | NA | NA | Dose 1:  1416769 (71.4%);  Dose 2:  743712 (78.3%) | Dose 1:  644492 (32.5%);  Dose 2:  569803 (60.0%) | Dose 1:  534248 (26.9%);  Dose 2:  504739 (53.2%) | Dose 1:  197725 (10.0%);  Dose 2:  357136 (37.6%) | NA |
| Sushila Kataria | India | Day 118±7 days post vaccination | HCWs | ChAdOx1  nCoV-19 | 2005 | NA | NA | NA | 1257 (62.7%); severe 5.1% | 670 (33.4%) | 870 (43.4%); severe 6.8% | 975 (48.4%); severe 7.2% | The analysis according to age reveals significantly higher levels among those in age group less than 50 years in comparison to those in age group more than 50 years. Combined for all commonly reported adverse reactions, the intensity was severe for 3.7%. |
| Si-Ho Kim | South Korea | After dose 1 | HCWs | ChAdOx1  nCoV-19 | 1403 | 35.84±11.13 | NA | 1301 (90.9%) | 1114 (77.8%) | 678 (47.4%) | 726 (50.7%) | 517 (36.1%) | 1015 (70.9%) need medication; 1 (0.1%) need hospitalization |
|  |  |  |  | BNT162b2 | 80 | 35.83±10.99 | NA | 42 (52.5%) | 41  (51.2%) | 6  (7.5%) | 6  (7.5%) | 4  (5.0%) | 6 (7.5%) need medication |
| Yao Rodion KONU | Togo | After dose 1 | HCWs | ChAdOx1  nCoV-19 | 1639 | Median 32 | NA | 1174 (71.6%) | 1068 (65.2%) | NA | 807 (49.2%) | 558 (34.1%) | Of the participants who experienced adverse events, 10.5% consulted a medical doctor, and 1.0% were hospitalized. |
| Cristina Menni | United Kingdom | 8 days after vaccination | GP | BNT162b2 (dose 1) | 282103 | Median 64 | 14369 (5.1%) with previous COVID-19 | Local: 150023 (71.9%) Systemic: 38155 (13·5%) | 61016 (29.2%) | 23674 (8.4%) | 21910 (7.8%) | 4236 (1.5%) | Most frequently reported effects occurred most often on the day after injection. Female, younger adults (aged <=55) and past SRAS-CoV-2 infection were associated with increased side effects. |
|  |  |  |  | BNT162b2 (dose 2) | 28207 | Median 59 | 2251 (8.0%) with previous COVID-19 | Local: 9025 (68.5%) Systemic: 6216 (22.0%) | 4515 (34.3%) | 4064 (14.4%) | 3731 (13.2%) | 1076 (3.8%) |  |
|  |  |  |  | ChAdOx1  nCoV-19 (dose 1) | 345280 | Median 65 | 14231 (4.1%) with previous COVID-19 | Local: 104282 (58.7%) Systemic: 116473 (33.7%) | 33939 (19.1%) | 72924 (21.1%) | 78734 (22.8%) | 28268 (8.2%) |  |
| Marco Montalti | Republic of San Marino | 7 days after vaccination | GP | Gam-COVID-Vac (dose 1) | 2558 | 18-89 | 1920 (75.1%) with underlying conditions; 93 (3.6%) with previous SARS-CoV-2 infection | Local: 261 (10.2%) Systemic: 660 (25.8%) | 634 (24.8%) | 609 (23.8%) | 473 (18.5%) | 304 (11.9%) | 20 (0.8%) were grade 3 and 8 (0.3%) grade 4. |
|  |  |  |  | Gam-COVID-Vac (dose 2) | 1288 | 18-89 | NA | Local: 207 (16.1%) Systemic: 238 (18.5%) | 564 (43.8%) | 411 (31.9%) | 282 (21.9%) | 200 (15.5%) | 17 (1.3%) grade 3 and 4 (0.3%) grade 4. |
| Joon Young Song | South Korea | 7 days after dose 1 | HCWs | ChAdOx1  nCoV-19 | 2426 | 19 and older | NA | NA | 2317 (95.5%) | 2126 (87.6%) | 1747 (72.0%) | 1556 (64.1%) | 2038 (84.0%) received antipyretics treatment |
|  |  |  |  | BNT162b2 | 52 | 19 and older | NA | NA | 49 (94.2%) | 28 (53.8%) | 15 (28.8%) | 9 (17.3%) | 11 (21.2%) received antipyretics treatment |
| Guangfei Wang | China | NA | HCWs | BBIBP-CorV | 4458 | NA | NA | NA | 93 (2.09%) | 22 (0.49) | 8 (0.18%) | 1 (0.02%) | 3 SAEs returned to normal with treatment. |
| Mei-Xian Zhang | China | NA | HCWs | CoronaVac (dose 1) | 1526 | 35.4 ± 8.9 | 85 (5.6%) with adverse reactions to other vaccines | 238 (15.6%) | NA | 127 (8.3%) | 92 (6.0%) | 45 (2.9%) | Female was associated with increase adverse reactions. |
|  |  |  |  | CoronaVac (dose 2) | 1397 | NA | NA | 204 (14.6%) | NA | 91 (6.5%) | 48 (3.4%) | 14 (1.0%) |  |

## Table S15. Sources of nationwide safety surveillance data

| **Country** | **Agency/Network** | **Link** |
| --- | --- | --- |
| Argentina | ESVAI | https://www.argentina.gob.ar/coronavirus/vacuna/equipos-salud/informes-seguridad |
| Austria | BASG | https://www.basg.gv.at/ueber-uns/covid-19-impfungen |
| Belgium | FAGG | https://www.fagg.be/nl/nieuws |
| Canada | CAEFISS, CANVAS | https://health-infobase.canada.ca/covid-19/vaccine-safety/ |
| Croatia | HALMED, HZJZ | https://www.halmed.hr/COVID-19/Kako-prijaviti-sumnju-na-nuspojavu/Podaci-o-zaprimljenim-prijavama-sumnji-na-nuspojave-cjepiva-protiv-bolesti-COVID-19/ |
| Czechia | SÜKL | https://www.sukl.cz/tydenni-zpravy-o-prijatych-hlasenich-podezreni-na-nezadouci  https://ourworldindata.org/covid-vaccinations |
| Denmark | Danish Medicines Agency | https://laegemiddelstyrelsen.dk/da/nyheder/temaer/Indberettede%20bivirkninger%20ved%20COVID-19%20vacciner/#total |
| Estonia | RAVIMIAMET | https://www.ravimiamet.ee/uudised |
| Finland | FIMEA | https://www.fimea.fi/web/en/about_us/coronavirus-covid-19-/adverse-reactions-reported-on-corona-vaccines |
| France | ANSM | https://ansm.sante.fr/actualites/?filter%5Bcategories%5D=22 |
| Germany | Paul-Ehrlich-Institut | https://www.pei.de/DE/newsroom/dossier/coronavirus/coronavirus-inhalt.html;jsessionid=FD48D0A1188B7CAD584F89ED9B1C5F65.intranet232?nn=169730&cms_pos=6 |
| Iceland | Icelandic Medicines Agency | https://www.lyfjastofnun.is/lyf/covid-19/aukaverkanatilkynningar-vegna-covid-19/  https://www.covid.is/tolulegar-upplysingar-boluefni |
| Ireland | HPRA | https://www.hpra.ie/homepage/medicines/safety-notices |
| Italy | AIFA | https://www.aifa.gov.it/ |
| Latvia | State Agency of Medicines | https://www.zva.gov.lv/lv/pacientiem-un-sabiedribai/zales/vakcinas-pret-covid-19/zinojumi-par-blaknem |
| Netherlands | Lareb Side Effects Center | https://www.lareb.nl/pages/wekelijkse-update-bijwerkingen |
| Norway | Norwegian Medicines Agency | https://legemiddelverket.no/godkjenning/koronavaksiner/meldte-mistenkte-bivirkninger-av-koronavaksiner |
| Poland | gov.pl | https://www.gov.pl/web/szczepimysie/raport-szczepien-przeciwko-covid-19 |
| Portugal | NFS | https://www.infarmed.pt/web/infarmed/relatorio |
| Singapore | Ministry of Health | https://www.moh.gov.sg/covid-19/vaccination |
| Slovakia | SUKL | https://www.sukl.sk/hlavna-stranka/slovenska-verzia/media/tlacove-spravy?page_id=2906 |
| Spain | Spanish Agency for Medicines and Healthcare Products (AEMPS) | https://www.aemps.gob.es/informa/boletines-aemps/boletin-fv/2021-boletin-fv/4o-informe-de-farmacovigilancia-sobre-vacunas-covid-19/ |
| Sweden | Swedish Medical Agency | https://www.lakemedelsverket.se/sv/coronavirus/coronavaccin/inrapporterade-misstankta-biverkningar---coronavacciner#hmainbody1 |
| Switzerland | Swissmedic | https://www.swissmedic.ch/swissmedic/en/home/news/coronavirus-covid-19/nebenwirkungen-covid-19-impfungen-update-5.html |
| United Kingdom | MHRA | https://www.gov.uk/government/publications/coronavirus-covid-19-vaccine-adverse-reactions/coronavirus-vaccine-summary-of-yellow-card-reporting |
| United States | VAERS, V-safe | https://vaers.hhs.gov/data/datasets.html?;  Shimabukuro TT, Cole M, Su JR. Reports of Anaphylaxis After Receipt of mRNA COVID-19 Vaccines in the US—December 14, 2020-January 18, 2021. *JAMA.* Published online February 12, 2021. doi:10.1001/jama.2021.1967;  Shay DK, Gee J, Su JR, et al. Safety Monitoring of the Janssen (Johnson & Johnson) COVID-19 Vaccine — United States, March–April 2021. MMWR Morb Mortal Wkly Rep 2021;70:680–684. DOI:http://dx.doi.org/10.15585/mmwr.mm7018e2;  COVID 19 vaccine safety update, Advisory Committee on Immunization Practices (ACIP). March 1, 2021 |

| Antonella d’Arminio Monforte | Italy | After each dose | HCWs | BNT162b2 | 3078 | >=16 | 396 (12.9%) with a history of COVID-19 | No previous COVID-19:  Dose 1: local reaction: 1481 (55.2%); systemic reaction: 789 (29.4%)  Dose 2: local reaction: 1675 (63.0%); systemic reaction: 1643 (61.8%)  Previous COVID-19:  Dose 1: local reaction: 274 (69.2%); systemic reaction: 206 (52.0%)  Dose 2: local reaction: 240 (61.2%); systemic reaction: 242 (61.7%) |
| --- | --- | --- | --- | --- | --- | --- | --- | --- |

## Table S16 Summary of COVID-19 vaccine safety surveillance data

| **Country** | **Vaccine/Manufacturer** | **Cut-off date** | **Doses administrated** | **Total AEFIs** | **SAEs** | **Note** |
| --- | --- | --- | --- | --- | --- | --- |
| Argentina‡ | Sputnik V | 2021/4/9 | 3,414,158 | 25,426 | 280 hospitalizations | - Fever with headache and/or myalgia and/or arthralgia (N=8,798) - Local pain and/or local reaction and/or local paresthesia and/or local lymphadenopathy (N=2,414) - Mild to moderate allergic reactions (N=399) - **Anaphylaxis (N=6)** - Gastrointestinal symptoms with or without fever (N=1,470) - Guillain Barre syndrome (Uncertain, N=2) - Anaphylaxis (Uncertain, N=2) |
|  | Oxford/AstraZeneca |  | 195,239 | 21 |  | - Fever with headache and/or myalgia and/or arthralgia and/or asthenia (N=8) - Local pain and/or local reaction and/or local paresthesia (N=6) |
|  | CoviShield |  | 587,816 | 2,048 |  | - Fever with headache and/or myalgia and/or arthralgia and/or asthenia (N=674) - Local pain and/or local reaction and/or local paresthesia (N=53) - Mild to moderate allergic reactions (N=25) - Odynophagia (N=5) - Gastrointestinal symptoms with or without fever (N=36) |
|  | Sinopharm |  | 1,295,940 | 410 |  | - Fever with headache and/or myalgia and/or arthralgia and/or asthenia (N=59) - Local pain and/or local reaction and/or local paresthesia and/or local lymphadenopathy (N=33) - Mild to moderate allergic reactions (N=65) - **Anaphylaxis (N=1)** - Gastrointestinal symptoms with or without fever (N=34) |
| Austria | Pfizer/BioNTech | 2021/5/28 | 3,495,168 | 7,210 | 92 death;  6 myocarditis;  69 life-threatening;  277 necessary or extended hospital stay. | - Among 119 reported deaths, 1 case’s connection with the vaccination is seen (death of the 49-year-old patient who died as a result of severe coagulation disorders); - 8 cases of suspected VITT were reported close to a vaccination with the COVID-19 vaccine from AstraZeneca (death of the 49-year-old patient who died as a result of severe coagulation disorders was also classified as VITT); - 11 cases of myocarditis have been reported close to a vaccination against COVID-19; - The side effects were reported as life-threatening in 123 patients; - In 563 patients a hospital stay was necessary or extended in connection with the COVID-19 vaccination. |
|  | Moderna |  | 507,987 | 1,645 | 11 death;  9 life-threatening;  46 necessary or extended hospital stay. |  |
|  | Oxford/AstraZeneca |  | 941,745 | 17,132 | 16 death;  8 VITT;  5 myocarditis;  44 life-threatening;  237 necessary or extended hospital stay. |  |
|  | Janssen |  | 36,004 | 98 | 1 life-threatening;  3 necessary or extended hospital stay. |  |
| Belgium | Pfizer/BioNTech | 2021/6/8 | 3,216,657 | 8,496 | 5258 | - 5258 (30%) among 17528 AEs were considered serious. |
|  | Moderna |  | 437,008 | 1,787 |  |  |
|  | Oxford/AstraZeneca |  | 1,348,696 | 7,078 |  |  |
|  | Janssen |  | 94,285 | 167 |  |  |
| Canada | Pfizer/BioNTech | 2021/6/4 | 18,894,651 | 3,763 | 896 | - 50 cases of TTS (Thrombosis with thrombocytopenia syndrome) were reported, 46 cases received COVISHIELD/AstraZeneca vaccines, 3 received a Pfizer vaccine, and 1 received a Moderna vaccine, among which 6 people died. - 35 cases of myocarditis/pericarditis were reported, 25 cases received Pfizer-BioNTech COVID-19 vaccine, 6 cases received Moderna vaccine, 3 cases received COVISHIELD/AstraZeneca vaccines. |
|  | Moderna |  | 5,096,282 | 2,151 | 176 |  |
|  | Oxford/AstraZeneca |  | 2,346,032 | 874 | 260 |  |
| Croatia | Pfizer/BioNTech | 2021/6/14 | 2,139,816 | 2,052 | 898 | Among the serious side effects, hypersensitivity reactions were most often observed. |
|  | Moderna |  |  | 353 |  |  |
|  | Oxford/AstraZeneca |  |  | 1468 |  |  |
|  | Janssen |  |  | 31 |  |  |
| Czechia | Pfizer/BioNTech, Moderna, Oxford/AstraZeneca | 2021/6/8 | 6,110,000 | 5,402 | NA | 78 deaths were reported due to suspected AEFIs. |
| Denmark | Pfizer/BioNTech | 2021/6/8 | 2,094,751 | 15,537 | NA | - 77 reports of severe allergic reactions were evaluated to be related to vaccination. 21 cases of anaphylaxis occurred after the first vaccination, and 12 cases of anaphylaxis occurred after the second vaccination; - In 108 of the 113 cases of blood clots, an association with the vaccine is considered less likely. In the remaining five cases, it cannot be ruled out that the cause is due to the needle itself. - 81 deaths were evaluated to be not related to vaccination. |
|  | Moderna |  | 185,169 | 2,510 | NA | - 4 reports of severe allergic reactions were evaluated to be related to vaccination; - In 18 cases of blood clots, an association with the vaccine is considered less likely. - 11 deaths were evaluated to be not related to vaccination. |
|  | Oxford/AstraZeneca |  | 150,694 | 23.236 | NA | - 23 reports of severe allergic reactions were evaluated to be related to vaccination. - 4 reports of blood clots cannot be excluded that the vaccine has been a contributory cause. - 2 deaths were evaluated to be not related to vaccination. |
|  | Janssen |  | 14,019 | 41 | NA | / |
| Estonia* | Pfizer/BioNTech | 2021/6/14 | 533,863 | 1,333 | NA | / |
|  | Moderna |  | 75,581 | 159 |  |  |
|  | Oxford/AstraZeneca |  | 203,897 | 2,486 |  |  |
|  | Janssen |  | 16,475 | 82 |  |  |
| Finland | Pfizer/BioNTech | 2021/6/9 | 2,587,708 | 1,533 | 813 | / |
|  | Moderna |  | 298,480 | 82 | 59 |  |
|  | Oxford/AstraZeneca |  | 406,100 | 855 | 559 |  |
| France | Pfizer/BioNTech | 2021/6/3 | 29,685,000 | 23,947 | 6,705 | / |
|  | Moderna |  | 3,492,000 | 3,540 | 743 | / |
|  | Oxford/AstraZeneca |  | 5,318,878 | 17,727 | 4,432 |  |
|  | Janssen |  | 336,038 | 80 | 34 |  |
| Germany | Pfizer/BioNTech | 2021/5/31 | 36,865,276 | 34,735 | 4,283 | / |
|  | Moderna |  | 3,972,764 | 8,319 | 380 |  |
|  | Oxford/AstraZeneca |  | 9,230,103 | 34,870 | 3,329 |  |
|  | Janssen |  | 472,941 | 733 | 14 |  |
| Iceland* | Pfizer/BioNTech | 2021/6/15 | 109,919 | 624 | 51 | / |
|  | Moderna |  | 18,502 | 296 | 12 |  |
|  | Oxford/AstraZeneca |  | 60,044 | 604 | 46 |  |
|  | Janssen |  | 35,726 | 125 | 3 |  |
| Ireland | Pfizer/BioNTech | 2021/5/13 | 1,922,913 | 3,734 | NA | / |
|  | Moderna |  |  |  |  |  |
|  | Oxford/AstraZeneca |  |  | 4,085 |  |  |
| Italy | Pfizer/BioNTech | 2021/5/26 | 22,285,723 | 47,631 | 4,011 | The most frequently reported vaccination-related serious adverse events are a flu-like syndrome with severe symptoms, more frequent after the second dose of mRNA vaccines and after the first dose of Vaxzevria.  The reporting rate of intracranial and atypical venous thrombosis in subjects vaccinated with Vaxzevria is in line with what has been observed at the European level (1 case per 100,000 first doses administered, no cases after second dose), mainly in people under 60 years of age. |
|  | Moderna |  | 2,901,137 | 2,564 | 377 |  |
|  | Oxford/AstraZeneca |  | 6,739,596 | 15,878 | 2,426 |  |
|  | Janssen |  | 503,155 | 171 | 23 |  |
| Latvia | Pfizer/BioNTech, Moderna, Oxford/AstraZeneca | 2021/6/13 | 947,038  (update to 6/6) | 1,615 | NA | / |
| Netherlands | Pfizer/BioNTech | 2021/6/6 | 7,300,000 | 111,852 | 980 | 159 reports of symptoms consistent with a severe allergic reaction, including 94 with pfizer/biontech vaccine (Comirnaty), 46 with the vaccine of AstraZeneca (Vaxzevria), 17 with the Vaccine of Moderna, 2 with the Vaccine of Janssen. In 43 reports, there was an established anaphylactic reaction. In the other reports, there were symptoms (such as extensive rashes or swelling around the eyes or throat) that may be appropriate for a severe allergic reaction. |
|  | Oxford/AstraZeneca |  | 1,300,000 | 145,423 | 251 |  |
|  | Moderna |  | 300,000 | 20,799 | 71 |  |
|  | Janssen |  | 9,000 | 606 | 0 |  |
| Norway | Pfizer/BioNTech | 2021/6/8 | 2,414,340 | 3302 | 167 deaths;  673 SAEs except deaths. | / |
|  | Moderna |  | 318,193 | 497 | 6 deaths;  110 SAEs except deaths. | / |
|  | Oxford/AstraZeneca |  | 261,624 | 6640 | 6 deaths;  414 SAEs except deaths. | / |
| Poland | Oxford/AstraZeneca, CureVac, Janssen, Moderna, Pfizer/BioNTech | 2021/6/15 | 24,687,629 | 10,941 | NA | / |
| Portugal | Pfizer/BioNTech | 2021/5/30 | 3,943,979 | 4,782 | 2,738 | 44 deaths were included in 2738 SAEs. Includes reports of cases of death occurring after vaccination with no direct causal relationship demonstrated with the vaccine administered. |
|  | Moderna |  | 521,683 | 387 |  |  |
|  | Oxford/AstraZeneca |  | 1,215,009 | 1,509 |  |  |
|  | Janssen |  | 109,409 | 17 |  |  |
| Singapore‡ | Pfizer/BioNTech, Moderna | 2021/4/18 | 2,213,888 | 2,796 | 95 | 95 reports (0.004% of administered doses) classified as serious AEs after assessment, of which there were 20 cases of anaphylaxis. |
| Slovakia | Pfizer/BioNTech | 2021/6/10 | 1,961,407 | 2,493 | 201 | 1 death was included in 201 SAEs. |
|  | Moderna |  | 296,050 | 559 | 37 | 1 death was included in 37 SAEs. |
|  | Oxford/AstraZeneca |  | 641,528 | 2,706 | 208 | 1 death was included in 208 SAEs, the cause of death determined by autopsy was related to thrombosis of the cerebral venous sinus, which is one of the possible rare side effects of the vaccine. |
| Spain | Pfizer/BioNTech* | 2021/3/21 | 4,834,876 | 23,084 | NA | / |
|  | Moderna |  | 304,715 | 2,741 | NA | / |
|  | Oxford/AstraZeneca |  | 985,528 | 6,343 | NA | 5 cases of venous breast thrombosis had been reported, two of them with associated thrombocytopenia, one of the cases had a fatal outcome. |
| Sweden | Pfizer/BioNTech* | 2021/6/10 | 4,568,479 | 15,789 | NA | / |
|  | Moderna |  | 597,293 | 4,475 | NA | / |
|  | Oxford/AstraZeneca |  | 886,815 | 21,891 | NA | / |
| Switzerland | Pfizer/BioNTech | 2021/5/4 | 2,800,000 | 870 | 701 | / |
|  | Moderna |  |  | 1,061 |  |  |
| United Kingdom | Oxford/AstraZeneca | 2021/6/2 | 40,200,000 | 717,250 | 725 anaphylaxes  863 deaths | 372 thrombo-embolic events with concurrent low platelets. |
|  | Moderna |  | 460,000 | 9,243 | 11 anaphylaxes  4 deaths | / |
|  | Pfizer/BioNTech |  | 25,400,000 | 193,768 | 395 anaphylaxes  406 deaths | / |
| United States | Pfizer/BioNTech | 2021/2/16 | 28,374,410 | 48,196 | 4,222 | Estimated anaphylaxis reporting rates was 4.7 per million doses admin (47/9,943,247, analytic period Dec 14-Jan 18). |
|  | Moderna |  | 26,738,383 | 56,567 | 1,859 | Estimated anaphylaxis reporting rates was 2.5 per million doses admin (19/7,581,429). |
|  | Janssen | 2021/5/7 | 7,980,000 | 13,725 | 343 | 17 events consistent with TTS, including three reports of non-CVST TTS (no deaths).  88 reports of death, three occurred in patients with CVST; after preliminary reviews, no other deaths appear to have an association with vaccination.  Among 79 reports of anaphylaxis after vaccination, four were confirmed as anaphylaxis cases after interview with a health care provider or review of medical records (<0.5 cases per 1 million doses administered); four reports remain under review. |

SAE: serious adverse events.

‡: Reported AEFIs were assessed as related to vaccination.

*: The number of doses administrated was actually the number of participants who received at least one dose of vaccine.

## Figure S1. Funnel plots to assess publication bias

1. **Location reaction**


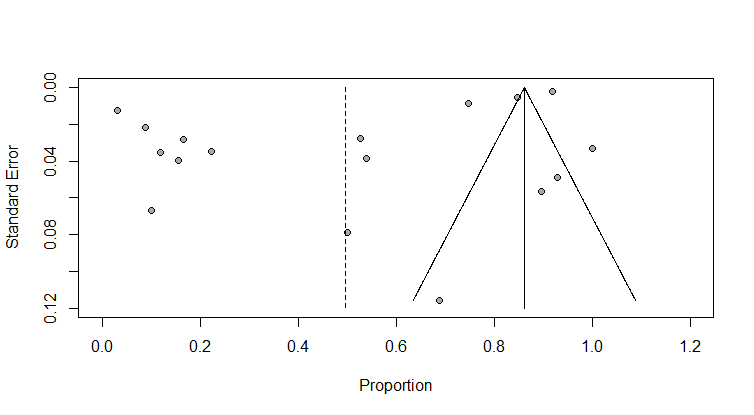


Rank correlation test of funnel plot asymmetry: p-value=0.32

1. **Systemic reaction**


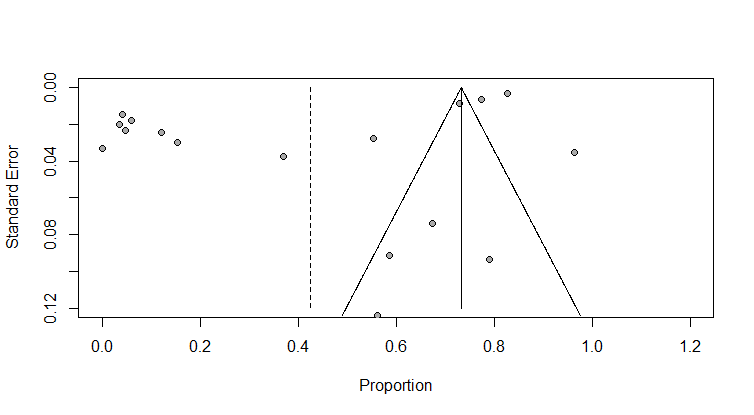


Rank correlation test of funnel plot asymmetry: p-value=0.28

1. Medically attended event


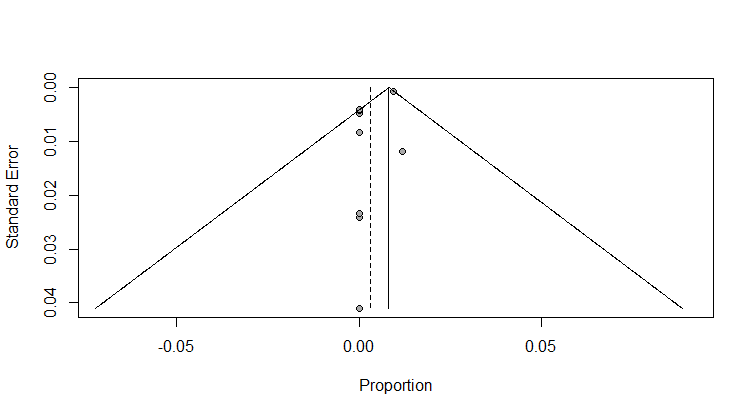


Rank correlation test of funnel plot asymmetry: p-value=0.06

## Figure S2. Forest plot of estimated results from meta-analysis of unsolicited adverse events by common system organ class (SOC).


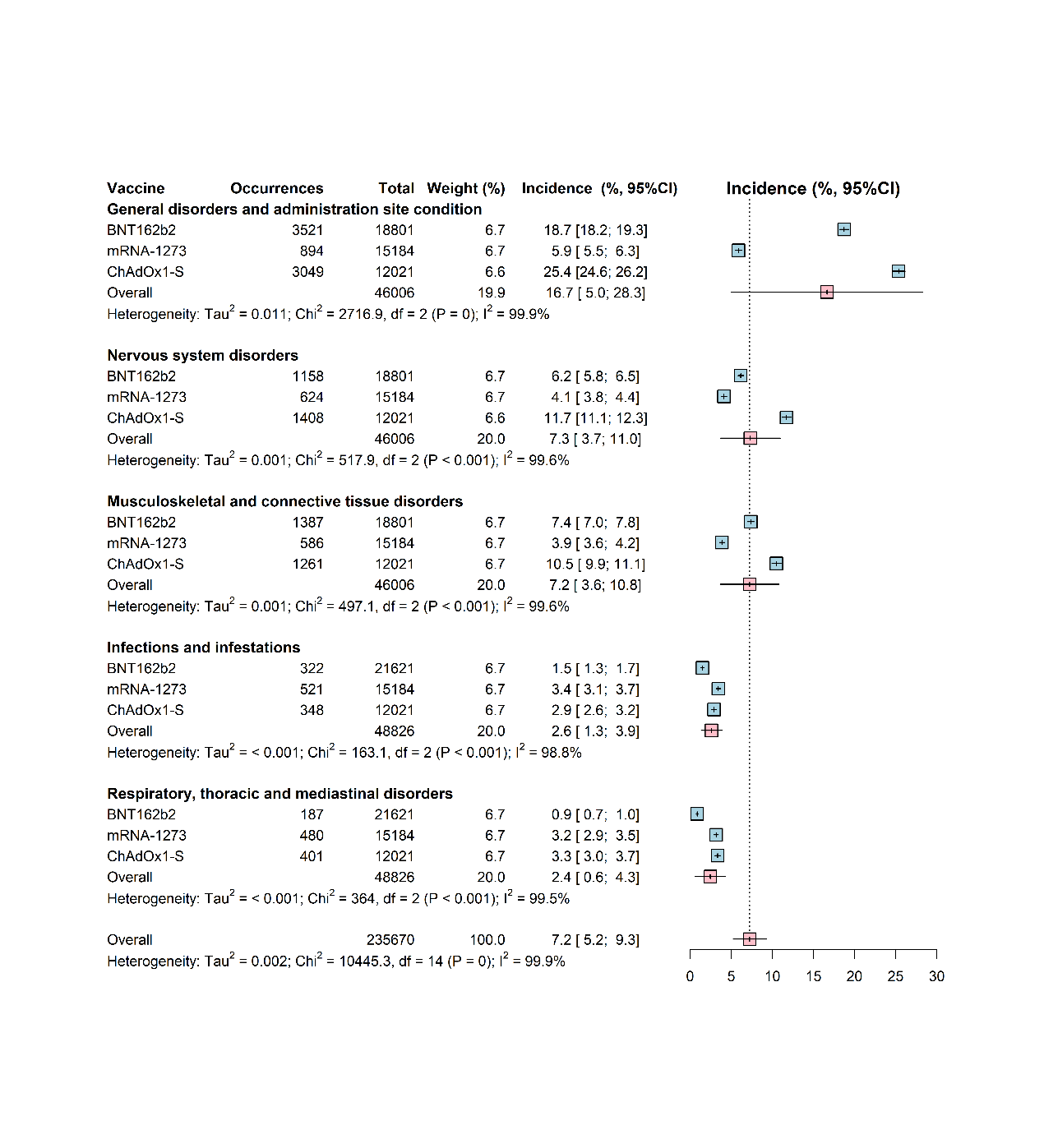


The size of the boxes represents the weight for each intervention group. The whisker represents the 95% confidence interval.

## Figure S3. Comparing rates of unsolicited adverse events by common system organ class (SOC) of COVID-19 vaccines versus placebos.


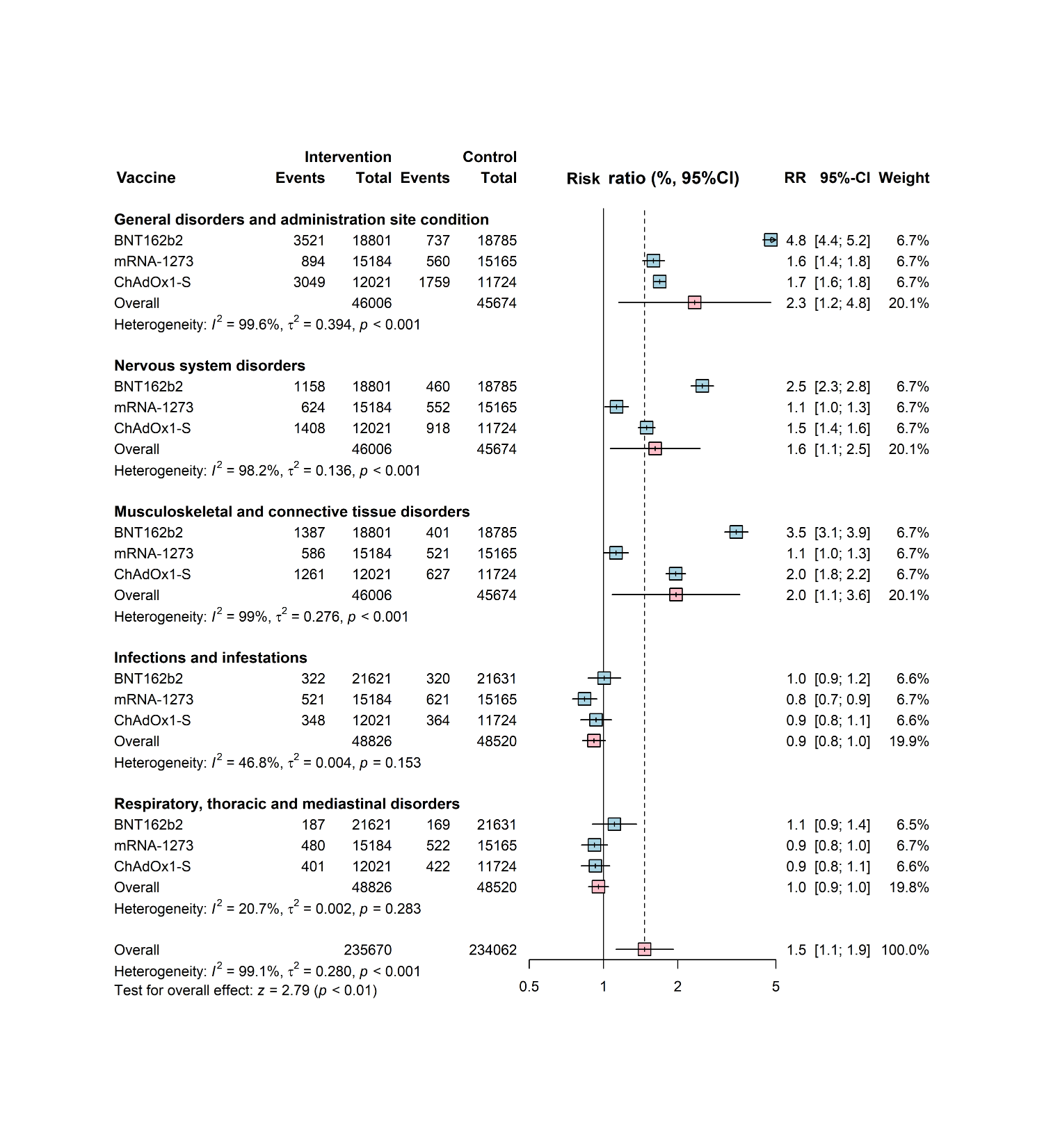


The size of the boxes represents the weight for each intervention group. The whisker represents the 95% confidence interval.

## Figure S4. Forest plot of estimated results from meta-analysis of local injection pain in adults from clinical trials


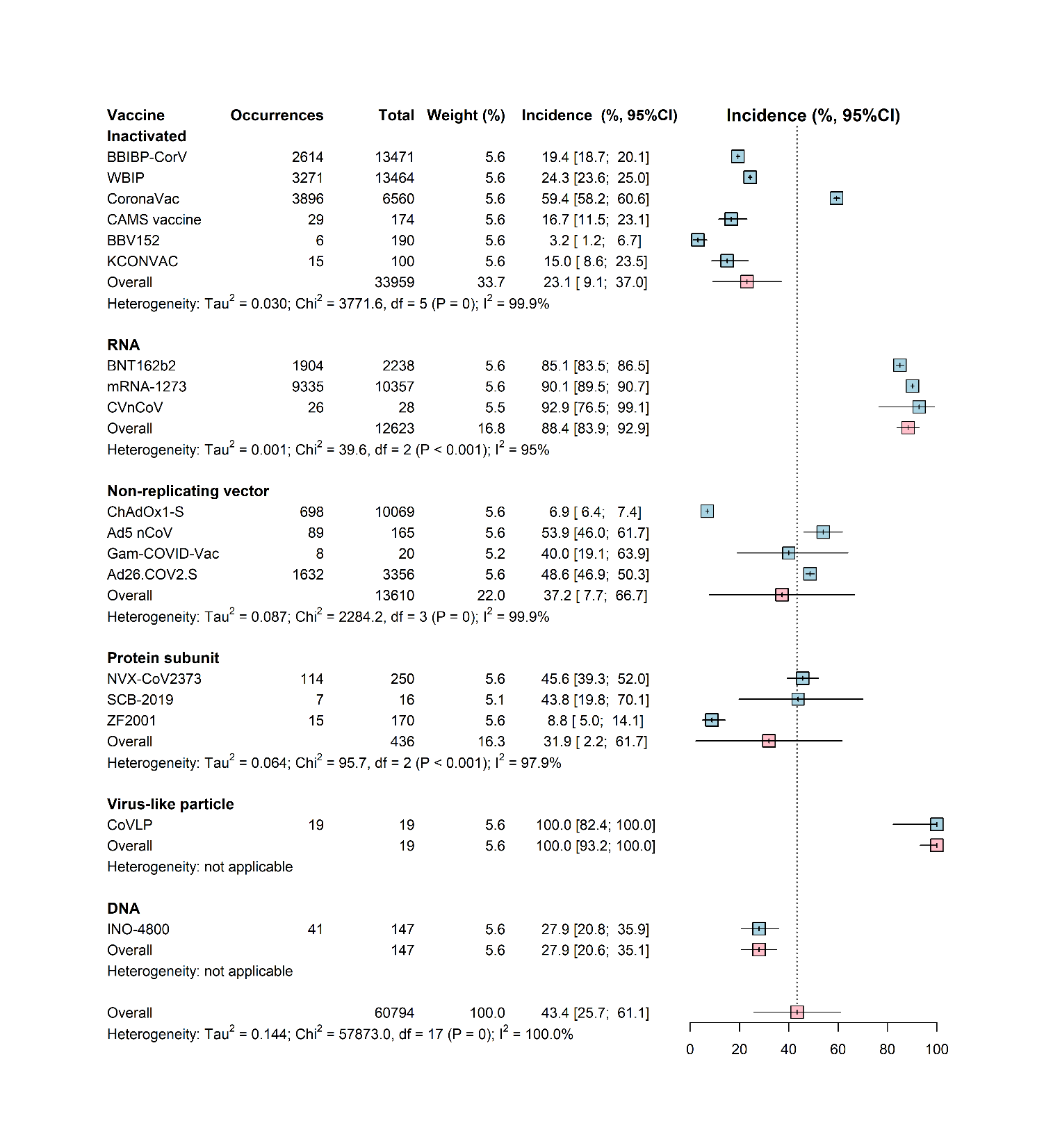


The size of the boxes represents the weight for each intervention group. The whisker represents the 95% confidence interval.

## Figure S5. Forest plot of estimated results from meta-analysis of fatigue in adults from clinical trials


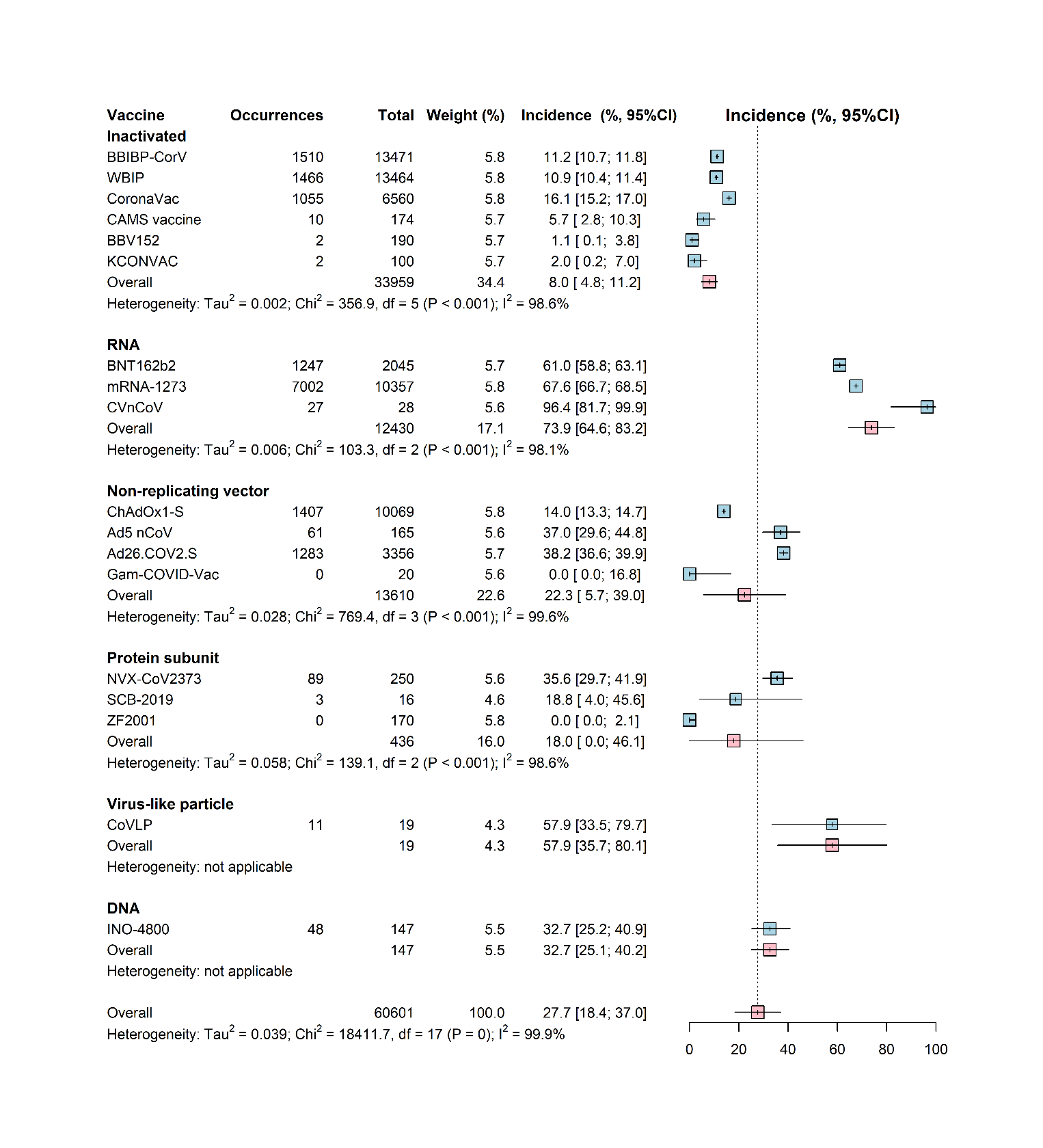


The size of the boxes represents the weight for each intervention group. The whisker represents the 95% confidence interval.

## Figure S6. Forest plot of estimated results from meta-analysis of headache in adults from clinical trials


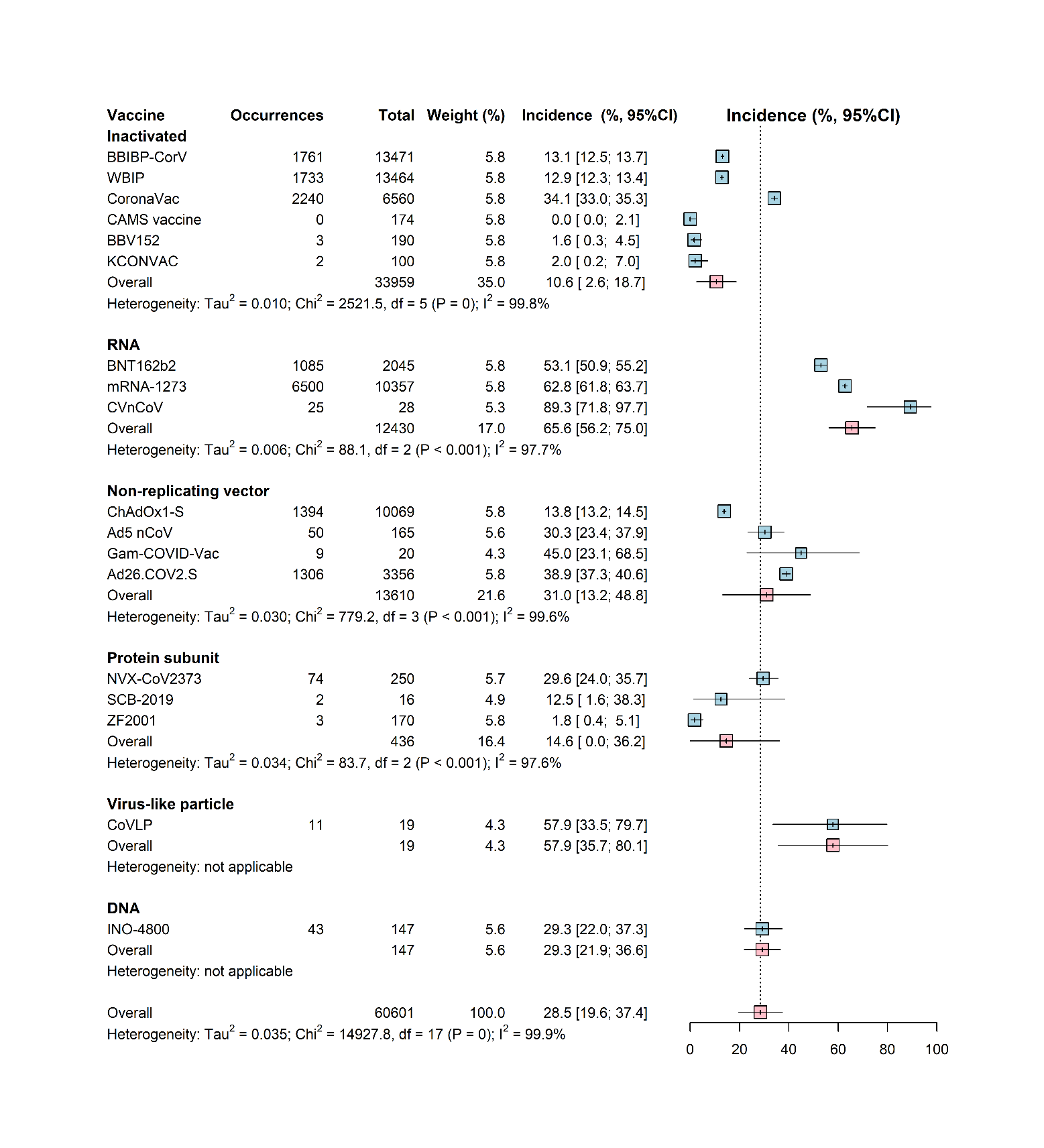


The size of the boxes represents the weight for each intervention group. The whisker represents the 95% confidence interval.

## Figure S7. Forest plot of estimated results from meta-analysis of fever in adults from clinical trials


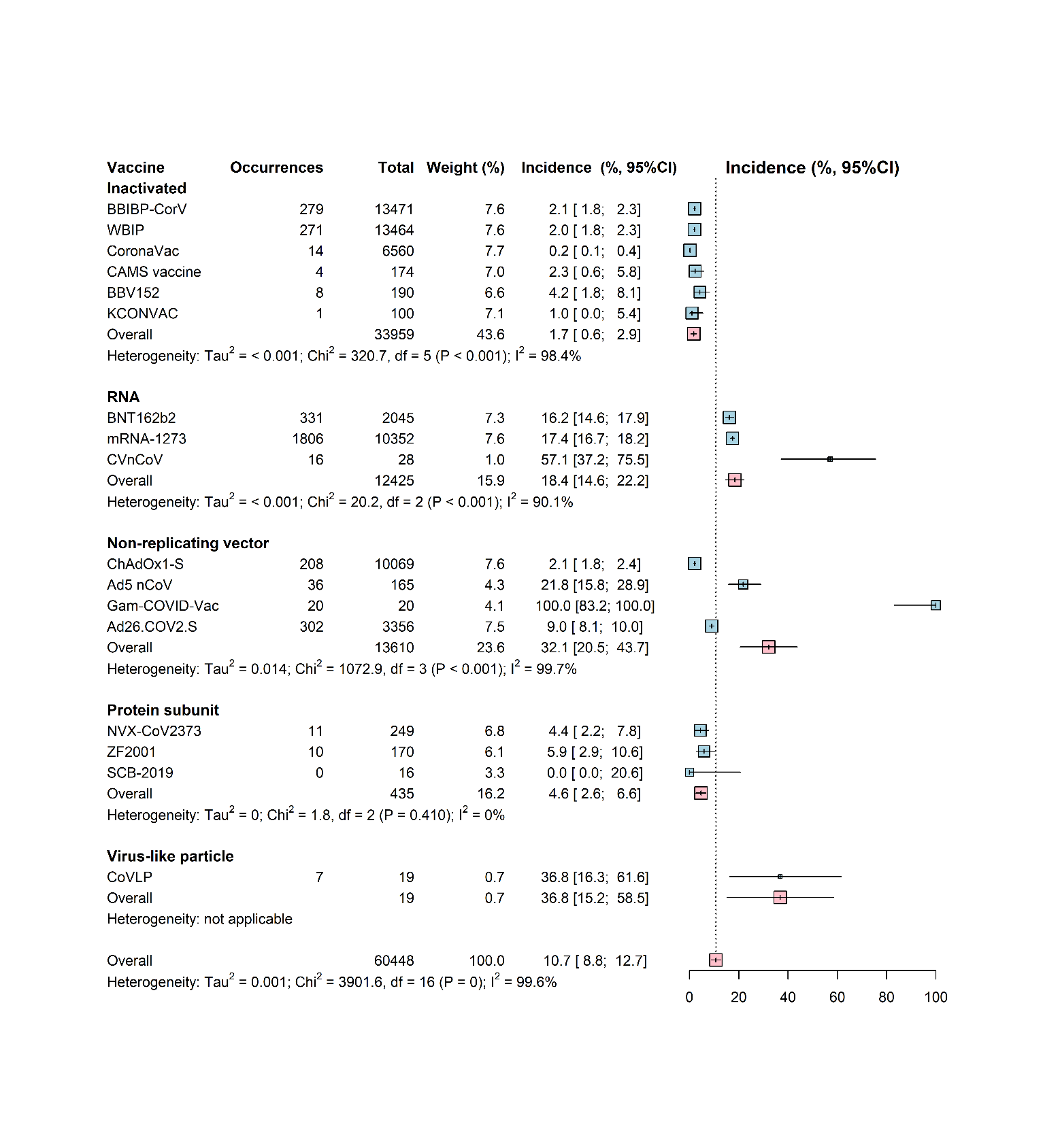


The size of the boxes represents the weight for each intervention group. The whisker represents the 95% confidence interval.
